# Antioxidant and Anti-Inflammatory Activities of Cytocompatible *Salvia officinalis* Extracts: A Comparison between Traditional and Soxhlet Extraction

**DOI:** 10.3390/antiox9111157

**Published:** 2020-11-20

**Authors:** Sara F. Vieira, Helena Ferreira, Nuno M. Neves

**Affiliations:** 1 3B’s Research Group, I3BS—Research Institute on Biomaterials, Biodegradables and Biomimetics, Headquarters of the European Institute of Excellence on Tissue Engineering and Regenerative Medicine, University of Minho, AvePark, Parque de Ciência e Tecnologia, Zona Industrial da Gandra, 4805-017 Barco, Guimarães, Portugal; sara.vieira@i3bs.uminho.pt (S.F.V.); helenaferreira@i3bs.uminho.pt (H.F.); 2 ICVS/3B’s–PT Government Associate Laboratory, Braga/Guimarães, Portugal

**Keywords:** *Salvia officinalis*, traditional extraction, soxhlet extraction, antioxidant activity, reactive oxygen and nitrogen species, anti-inflammatory activity, cytocompatibility

## Abstract

Chronic inflammation is characterized by an overproduction of several inflammatory mediators (e.g., reactive species and interleukins -IL) that play a central role in numerous diseases. The available therapies are often associated with serious side effects and, consequently, the need for safer drugs is of utmost importance. A plant traditionally used in the treatment of inflammatory conditions is *Salvia officinalis*. Therefore, conventional maceration and infusion of its leaves were performed to obtain hydroethanolic (HE-T) and aqueous extracts (AE-T), respectively. Their efficacy was compared to soxhlet extracts, namely aqueous (AE-S), hydroethanolic (HE-S), and ethanolic extracts (EE-S). Thin-layer chromatography demonstrated the presence of rosmarinic acid, carnosol, and/or carnosic acid in the different extracts. Generally, soxhlet provided extracts with higher antioxidant activities than traditional extraction. Moreover, under an inflammatory scenario, EE-S were the most effective, followed by HE-S, HE-T, AE-T, and AE-S, in the reduction of IL-6 and TNF-α production. Interestingly, the extracts presented higher or similar anti-inflammatory activity than diclofenac, salicylic acid, and celecoxib. In conclusion, the extraction method and the solvents of extraction influenced the antioxidant activity, but mainly the anti-inflammatory activity of the extracts. Therefore, this natural resource can enable the development of effective treatments for oxidative stress and inflammatory diseases.

## 1. Introduction

Inflammation is essential for human life, leading to the elimination of noxious stimuli and restoration of tissue homeostasis [1]. However, if the inflammatory response is prolonged, continuous recruitment of inflammatory cells and overproduction of reactive oxygen and nitrogen species (ROS/RNS) are observed, inducing tissue damage [1,2,3].

High amounts of ROS/RNS (e.g., superoxide radical anion -O_2_^•−^- and nitric oxide -^•^NO) are produced by activated immune cells (e.g., neutrophils and macrophages) to protect the organism against, for instance, bacteria or intracellular parasites [4,5]. ROS/RNS can also activate several essential inflammatory pathways, which will result in the release of several proinflammatory cytokines (e.g., interleukin -IL-6, tumor necrosis factor -TNF-α) and chemokines (e.g., IL-8) with the ability to recruit more immune cells to the site of inflammation [6,7]. IL-6 is a particularly important mediator of the acute phase response, inducing fever, stimulating the production of neutrophils in the bone marrow, and supporting the growth and differentiation of B cells [8]. Another cytokine with an important role in the pathogenesis of inflammatory conditions is TNF-α to regulate cell growth and proliferation, the release of adhesion molecules, and the expression of inflammatory mediators [9].

Unfortunately, high amounts of ROS/RNS and proinflammatory mediators in the tissues/cells can irreversibly damage several biomolecules and/or cells, resulting in the loss of their function and/or death. For instance, ROS/RNS can induce lipid peroxidation, leading to the cells’ membrane damage, culminating with their death [10]. Consequently, a dysregulated inflammatory response can lead to several diseases, such as rheumatoid arthritis, osteoarthritis, cardiovascular diseases, neurodegenerative diseases, or cancer [7,11,12,13]. Therefore, the administration of antioxidant and/or anti-inflammatory compounds is crucial to stop the unregulated inflammatory scenario. Currently available therapies are based on nonsteroidal anti-inflammatory drugs (NSAIDs, e.g., diclofenac, salicylic acid, and celecoxib), corticosteroids (e.g., dexamethasone and betamethasone), conventional disease-modifying anti-rheumatic drugs (DMARDs, e.g., methotrexate), and biological agents (e.g., anti-TNF-α and anti-IL-1β antibodies) [14,15,16]. These therapeutic agents, however, are often associated with several serious side effects [17,18,19,20,21,22,23]. Therefore, the need to discover new, safe, and effective anti-inflammatory drugs is of utmost importance.

Plants have been the basis of traditional medicine in many cultures for thousands of years. Even nowadays, plants, being a rich source of bioactive molecules, are widely used by the world’s population to treat several diseases [24]. Moreover, more than one hundred compounds in clinical use are derived from plants [25,26,27]. Some examples of plant-derived drugs widely prescribed include morphine (analgesic, *Papaver somniferum*), digitoxin (cardiotonic, *Digitalis purpurea*), and vincristine (anti-cancer, *Catharanthus roseus*). Indeed, there is an increasing interest in drug discovery from plant origin [28,29], mainly due to the reduction of the costs associated with the development of new drugs [30,31].

*Salvia officinalis* (Lamiaceae family), commonly known as garden sage, Dalmatian sage, or common sage, is widely used in traditional medicine. An infusion of dried sage leaves with boiling water (sage tea) has been traditionally used to treat mouth, throat, and bronchial inflammations, as well as coughs and stomach pain [32,33]. Sage leaves can also be directly applied for cold sores, gum disease, sore mouth, throat or tongue, and swollen tonsils. Particularly in inflammatory diseases, different extracts of *S. officinalis* demonstrated the ability to reduce IL-6 and IL-8 production [34], inhibit ^•^NO generation [35], increase the number of antioxidant enzymes [36], and reduce leukocyte infiltration, plasmatic extravasation, and edema [37]. However, the properties of tea and tinctures of *S. officinalis* on the behavior of human macrophages have not been reported in detail, in spite of its common use in inflammation treatment. In this work, traditional extraction, namely maceration and infusion, was performed to obtain aqueous (AE-T) and hydroethanolic extracts (HE-T), respectively. To increase the yield of extraction, aqueous (AE-S), hydroethanolic (HE-S), and ethanolic extracts (EE-S) were prepared using a soxhlet. After extraction, the total phenol and flavonoid content (TPC and TFC, respectively) were measured, and the composition of *S. officinalis* extracts was analyzed by thin-layer chromatography (TLC). The antiradical activity of *S. officinalis* extracts against 2,2-diphenyl-1-picrylhydrazyl (DDPH^•^) and 2,2′-azino-bis(3-ethylbenzothiazoline-6-sulfonic acid) diammonium salt (ABTS^•+^) was also evaluated, as well as their antioxidant activity against the most powerful ROS/RNS, namely ROO^•^, O_2_^•−^, and ^•^NO. The reducing power (RP) of the five *S. officinalis* extracts was also investigated. The cytotoxicity of *S. officinalis* extracts was evaluated using the L929 cell line, according to ISO 10993-5:2009 [38]. Moreover, their capacity to stimulate or inhibit the production of two proinflammatory cytokines with a crucial role in the inflammatory response, IL-6 and TNF-α, was investigated using nonstimulated or LPS-stimulated macrophages (THP-1 cell line), respectively. For all cellular studies, the metabolic activity, the DNA concentration, the total protein content, and the cells’ morphology were analyzed.

To the best of our knowledge, this is the first study that exhaustively compares the efficiency of several *S. officinalis* extracts obtained from soxhlet and traditional extraction regarding their cytocompatibility and antioxidant and anti-inflammatory activities.

## 2. Materials and Methods

### 2.1. Reagents

An aluminum TLC plate, silica gel coated with fluorescent indicator F254 (20 × 20 cm), chloroform, ethyl acetate, acetic acid, ethanol, sodium carbonate (Na_2_CO_3_), Folin–Ciocalteu’s phenol reagent, gallic acid, aluminum chloride, rutin, DPPH^•^, ABTS^•+^, potassium phosphate dibasic, potassium phosphate monobasic, fluorescein sodium salt, 2,2′-azobis(2-methylpropionamidine) dihydrochloride (AAPH), sodium nitroprusside dihydrate (SNP), sulfanilamide (SA), N-(1-Naphthyl)ethylenediamine dihydrochloride (NED), phosphoric acid, phosphate-buffered saline (PBS), β-Nicotinamide adenine dinucleotide (NADH), nitrotetrazolium blue chloride (NBT), phenazine methosulfate (PMS), sodium phosphate dibasic, sodium phosphate monobasic, potassium ferricyanide (III), trichloroacetic acid and ferric chloride (III), low-glucose Dulbecco’s modified eagle’s medium (DMEM), lipopolysaccharide (LPS) (Escherichia coli O26:B6), dexamethasone, diclofenac, and salicylic acid were purchased from Sigma. High-purity standards, including rosmarinic acid, α-terpineol, 1,8-cineole, apigenin 7-glucoside, salvianolic acid B, ursolic acid, cinnamic acid, quinic acid, linalyl acetate, viridiflorol, apigenin, carnosol, betulinic acid, oleanolic acid, α-humulene, (−)-camphor, (+)-borneol, protocatechuic acid, luteolin, camphene, luteolin 7-glucoside, isoquercitrin, p-coumaric acid, apigenin 7-O-glucuronide, linalool, α-pinene, carnosic acid, eriocitrin, ferulic acid, and quercetin, were also obtained from Sigma. Celecoxib was obtained from abcr GmbH. CellTiter 96^®^ AQueous One Solution Cell Proliferation Assay was obtained from Promega, while dimethyl sulfoxide (DMSO) was purchased from VWR. Formic acid was obtained from PanReac AppliChem. Fetal bovine serum (FBS) and antibiotic/antimycotic solution, TrypLE Express, Roswell Park Memorial Institute (RPMI) 1640 medium, Quant-iT PicoGreen dsDNA Kit, and Micro BCA protein assay kit were obtained from Thermo Fisher Scientific. AlamarBlue^®^ was acquired from Bio-Rad. Human IL-6 and TNF-α DuoSet enzyme-linked immunosorbent assay (ELISA) and DuoSet ELISA Ancillary Reagent Kit 2 were purchased from R&D Systems.

### 2.2. Bioactive Compounds Extraction

*S. officinalis* was purchased from Cantinho das Aromáticas (Porto, Portugal) and grown in an organic environment. After one year of cultivation, the leaves, collected in May before the blooming of the flowers, were dried in the dark at room temperature (RT). The dried leaves were then cut into small pieces and stored in the same conditions until the preparation of the extracts.

To mimic the extracts traditionally prepared by infusion (AE-T) and maceration (HE-T), 200 mL of boiling water or 100 mL of hydroethanolic solution (50:50) was added to 10 g of *S. officinalis* leaves for 10 min or 5 days, macerating 1 time per day, at RT, respectively. A soxhlet apparatus was also used to prepare AE-S, HE-S, and EE-S from 10 g of *S. officinalis* leaves. After each extraction, the solvent was replaced with a new solvent to prevent *S. officinalis* extracts from being exposed to high temperatures, which could result in the thermal decomposition of thermolabile compounds [39]. All extracts were filtrated through a 0.45 µm filter and frozen at −80 °C and lyophilized (LyoAlfa 10/15, Telstar Technologies, S-L, Terrassa, Spain. Before the freeze-drying of HE, ethanol was evaporated at RT under reduced pressure using a rotary evaporator (IKA VACSTAR D S099, IKA^®^ - Werke GmbH & Co.KG, Staufen im Breisgau, Germany). This last procedure was used to obtain dried EE-S. After that, the yield extraction in percentage (%) was calculated by dividing the dry weight of the extracts by the initial weight of leaves. The extracts were stored at −20 °C until further use. Three different batches of each extract were prepared.

### 2.3. Determination of the TPC

TPC of the *S. officinalis* extracts was measured using the Folin–Ciocalteu method, according to the procedure described by Kontogianni et al. with some modifications [40]. All the extracts (1 mg/mL) were dissolved in their solvent of extraction (water, ethanol, or their 50:50 mixture). Then, 200 µL of the extract was mixed with 4.8 mL of distilled water and 500 µL of Folin–Ciocalteu reagent. After 3 min, 1 mL saturated solution of Na_2_CO_3_ (332 g/L) was added and diluted with distilled water to 10 mL. Blank samples were prepared using 200 µL of the respective solvent without *S. officinalis* extracts. After 1 h, a volume of 200 µL was pipetted to a 96-well plate, and the absorbance was read at 725 nm in a microplate reader (Synergy^TM^ HT Multi-Mode Microplate Reader, BioTek, Winooski, VT, USA). Gallic acid (GA), in concentrations ranging from 25 to 500 mg/mL, was used to prepare the calibration curve to interpolate the content of phenols of each extract. The results are expressed as mg of GA per g of dry *S. officinalis* extract. All measurements were performed in triplicate for each batch of each extract.

### 2.4. Determination of the TFC

TFC was determined by the aluminum chloride colorimetric method, according to the procedure of Kontogianni et al. with some modifications [40]. One milliliter of *S. officinalis* extracts (10 mg/mL) dissolved in their solvent of extraction was mixed with 1 mL of aluminum chloride in ethanol (20 mg/mL) and diluted with ethanol to 25 mL. Blank samples were prepared with 1 mL of each extract mixed with 1 drop of acetic acid and diluted to 25 mL. After 40 min of incubation at RT, 200 µL of the resulting solution was pipetted to a 96-well plate, and the absorbance was read at 415 nm at 20 °C in a microplate reader (Synergy^TM^ HT Multi-Mode Microplate Reader, BioTek, Winooski, VT, USA). Rutin (R), in concentrations ranging from 25 to 500 mg/mL, was used to prepare the calibration curve to interpolate the content of flavonoids. The results are expressed as mg of R per g of dry *S. officinalis* extract. All measurements were performed in triplicate for each batch of each extract.

### 2.5. Chromatographic Analyses

Thin-layer chromatography (TLC) was conducted, in triplicate, according to the procedure described by Exarchou et al. [41]. After application of the samples and standards dissolved in methanol in the plates, they were developed with a mobile phase consisting of chloroform, ethyl acetate, and formic acid (5:4:1, *v*/*v*/*v*) in an ascending one-dimensional mode in a saturated glass chamber. After separation and drying the plates, the separated compounds were revealed using iodine and UV light (254 nm). The retention factor (R_F_) of each compound in the samples was calculated and compared with the R_F_ of the standards. Afterwards, samples and standards were mixed at the same point of application to demonstrate the presence of that phytochemical compound, since small differences in the R_F_ values are detected by this test.

### 2.6. Preparation of S. officinalis Extracts Solutions and IC_50_ Calculation for Antioxidant Activity Assays

Stock solutions of *S. officinalis* extracts were prepared at concentrations of 500 ug/mL in the respective buffer of each assay. AE, HE, and EE were dissolved in 25% of their solvent of extraction. *S. officinalis* extracts stock solutions were then serially diluted to obtain final concentrations of 5, 10, 25, 75, 125, and 250 μg/mL. For all assays, control samples without the extracts but with an equal volume of buffer were also prepared. A control with 25% of the extraction solvent was also performed to demonstrate its noninterference in the antioxidant activity assessment. The assays were performed in triplicate for each batch of each extract. A microplate reader (Synergy^TM^ HT Multi-Mode Microplate Reader, BioTek, Vermont, USA) was used to read either the absorbance or the fluorescence. In all antioxidant assays, the extract concentration required to inhibit 50% of the radical (half-maximal inhibitory concentration (IC_50_)), in μg/mL, was calculated by linear or nonlinear regression of the plots presenting the extract concentration (μg/mL, in abscissa) vs. the average (%, in ordinate) of the respective radical in the solution. Lower IC_50_ values mean the higher ability of *S. officinalis* extracts to neutralize the studied radicals.

#### 2.6.1. DPPH^●^ Radical Scavenging Activity

The ability of the *S. officinalis* extracts to neutralize DPPH^●^ was determined according to the method described by Cidade et al. [42]. The concentration of 1.9 mM DPPH^●^ ethanolic solution was adjusted with ethanol in a microplate reader to obtain an absorbance of 0.38 ± 0.01 at 515 nm (25 °C) for 180 µL of the radical solution. Then, 20 µL of each *S. officinalis* extract at different concentrations in ethanol (Section 2.6) were added to a 96-well plate, and 180 µL of DPPH^●^ ethanolic solution was mixed. The absorbance was immediately recorded at 515 nm every minute for 60 min at 25 °C. The neutralization of this radical is accompanied by discoloration of the solution, from deep purple to yellow [43]. Equation (1) was used to calculate the percentage of the DPPH^●^ in the solution over time:

(1) $${DPPH}^{•}\;\left(\%\right)=\frac{abs_{t\;=\;x}}{abs_{t\;=\;0}}\;\times 100$$

where *abs_t_*_=*x*_ and *abs_t_*_=0_ is the absorbance of the mixture at a given time and initial absorbance, respectively.

#### 2.6.2. ABTS^●+^ Radical Scavenging Activity

The ability of the *S. officinalis* extracts to scavenge ABTS^●+^ was determined according to the method described by Re et al. with slight modifications [44]. The monocation radical ABTS^●+^ was generated by the reaction between ABTS (7 mM) with the oxidizing agent potassium persulfate (2.45 mM) in an aqueous solution, for 12–16 h, at RT protected from the light. The ABTS^●+^ radical concentration was adjusted with ethanol to an absorbance of 0.45 ± 0.01 at 734 nm for each 180 µL of a radical solution, in a microplate reader, at 30 °C. Then, 20 µL of each *S. officinalis* extracts at different concentrations in ethanol (Section 2.6) were added to a 96-well plate, and 180 µL of ABTS^●+^ ethanolic solution was mixed. The decrease in the absorbance was immediately recorded at 734 nm every minute for 30 min, at 30 °C. ABTS^●+^ neutralization is accompanied by discoloration of solution from blue to green. The percentage of the ABTS^●+^ in the solution over time was calculated using Equation (1).

#### 2.6.3. Antioxidant Activity against ROO^●^

The antioxidant activity of *S. officinalis* extracts against ROO^●^ was evaluated according to a method reported in the literature with some modifications [45]. *S. officinalis* extracts were prepared as described in Section 2.6, but, in this assay, the final concentrations were 0.5, 1, 2, 3, 4, 5, and 10 μg/mL. Then, 150 µL *S. officinalis* extracts at different concentrations in 75 mM potassium phosphate buffer (pH 7.4) were incubated with 25 µL of the fluorescent probe fluorescein (final concentration 48 nM). ROO^●^ were generated by the thermodecomposition of the water-soluble initiator AAPH (25 µL, final concentration of 15 mM) [46]. The fluorescence was immediately recorded with an excitation wavelength of 485 nm and an emission wavelength of 528 nm, at 37 °C, every minute for 3 h in a microplate reader. A decrease in fluorescence intensity means that the fluorescein was oxidized by ROO^●^ [46]. Data obtained were converted to relative fluorescence values by dividing the fluorescence intensity at a given time by the fluorescent intensity at 0 min. The antioxidant capacity was calculated for each *S. officinalis* extract concentration by the area under the curve (AUC), using Equation (2):

(2) $$Antioxidant\;Capacity\;=\;\frac{\;AUC_{extracts}-AUC_{blk}}{AUC_{blk}}\times 100^{\;}$$

where *AUC_extract_* corresponds to the AUC obtained for a given concentration of *S. officinalis* extracts, whereas *AUC_blk_* is related to the AUC in the absence of extracts (blank, 0 μg/mL). The AUC was calculated by integrating the relative fluorescence curve as a function of the time using GraphPad Prism 6 software.

#### 2.6.4. Antioxidant Activity against ^●^NO

The antioxidant activity of different *S. officinalis* extracts against ^●^NO was measured using Griess reagent, according to the method of Pardau et al. [47]. In a 96-well plate, 20 µL of each *S. officinalis* extracts at different concentrations (Section 2.6) and 80 µL SNP (10 mM) in PBS were mixed. The plate was incubated for 15 min in a water bath at 37 °C under a tungsten light [48]. In these conditions (physiological pH and presence of light), SNP reacts spontaneously with oxygen, generating ^●^NO [49]. ^●^NO also immediately interacts with oxygen to form nitrogen dioxide (NO_2_), which, in turn, reacts with ^●^NO, generating dinitrogen trioxide (N_2_O_3_) [50]. In the presence of water, N_2_O_3_ can generate nitrite ions (NO_2_^−^), which can be quantified using the Griess reagent. The addition of 1% SA in 20% acetic acid (50 µL) led to its diazotization in the presence of NO_2_^−^. After 10 min, 0.1% NED in 2.5% phosphoric acid (50 µL) was added to produce a stable water-soluble pink azo dye, whose absorbance was measured at 540 nm. A ^●^NO scavenger competes with oxygen, donating protons to NO_2_^−^, reducing its production, and, therefore, the absorbance, which can be used as an indirect indicator of ^●^NO concentration. The percentage of nitrite in the solution was determined according to the followed Equation (3):

(3) $${\mathrm{NO}}_{2}^{-}{\;\mathrm{or}\;O}_{2}^{•-}\;\left(\%\right)=\frac{{\mathrm{abs}}_{\mathrm{extract}}}{{\mathrm{abs}}_{\mathrm{control}}}\;\times 100$$

where abs_control_ is the absorbance of the control (0 μg/mL) and abs_extract_ is the absorbance in the presence of *S. officinalis* extracts at different concentrations.

#### 2.6.5. Antioxidant Activity against O_2_^●−^

A method from Fernandes et al. was used to determine the antioxidant activity against O_2_^●−^ [48]. In the presence of oxygen, O_2_^●−^ were generated by the NADH/PMS system, which will reduce the NBT to a blue chromogen [48]. This could be prevented in the presence of an antioxidant. In a 96-well plate, 26.1 µL of *S. officinalis* extracts at different concentrations in PBS (Section 2.6), 75 µL of NADH (final concentration 166 µM), 150 µL of NBT (final concentration 43 µM), and 10 µL of PMS (final concentration of 2.7 µM) were mixed. The absorbance was immediately recorded at 560 nm for 2 min at RT. The percentage of O_2_^●−^ in the solution was determined according to Equation (3).

#### 2.6.6. RP Capacity

The capacity of the *S. officinalis* extracts to convert Fe^3+^ into Fe^2+^ (reducing power (RP)) was investigated according to a method described by Martins et al. with some modifications [43]. To each *S. officinalis* extract at different concentrations (500 µL) in 200 mM sodium phosphate buffer, pH 6.6, (Section 2.6) were added 500 µL sodium phosphate buffer and 500 µL ferricyanide (1% *w*/*v*). The mixture solution was incubated at 50 °C for 20 min. Then, 500 µL trichloroacetic acid (10% *w*/*v*) was added. Antioxidant species reduce the ferrocyanide reagent [K_4_Fe(CN)_6_], forming K_3_Fe(CN)_6_. From this solution, 114 µL were removed and added to a 96-well plate, together with 114 µL of deionized water and 23 µL of ferric chloride (0.1% *w*/*v*). The mixtures were homogenized and left at RT for 4 h. The ferrous amount could then be determined by the formation of Peal’s Prussian blue: the addition of ferric chloride (FeCl_3_) allowed the generation of a blue-colored product (KFe[Fe(CN)_6_]) that could be determined spectrophotometrically at 690 nm. An increase in the absorbance indicates an increase in the reduction of Fe^3+^ into Fe^2+^ by *S. officinalis* extracts. In this assay, the IC_50_ is the extract concentration that provides a 0.5 absorbance. Higher IC_50_ values correspond to the higher ability of *S. officinalis* extracts to convert Fe^3+^ into Fe^2+^.

### 2.7. Preparation of S. officinalis Extracts Solution for Biological Studies

For cytotoxic assays, HE and EE were firstly dissolved in 0.4% and 1% DMSO, respectively. DMEM was then added to obtain a stock solution of 250 μg/mL. For immunomodulatory assays, aliquots of the stock solutions (12.8 mg/mL for AE and 60.0 mg/mL for HE and EE) of each *S. officinalis* extract were also prepared and stored at −80 °C. AE were completed dissolved in complete RPMI medium, while EE and DE were dissolved in DMSO. The percentage of DMSO in the well was 0.33% for the maximal concentration tested of HE and EE. Then, both stock solutions were sterilized with a 0.22 µm filter and diluted to final concentrations of 5, 10, 25, 75, 125, and 250 μg/mL in the respective medium. A DMSO screening was performed before, and the metabolic activity was not affected by the percentages used.

### 2.8. Cytotoxicity Evaluation

#### 2.8.1. Cell Culture and Seeding

The cytotoxic effect of *S. officinalis* extracts in the presence of the mouse adipose fibroblast cell line (L929) was evaluated according to a procedure described by Vieira et al. with some modifications [51]. The L929 cell line was cultured in low-glucose DMEM supplemented with 10% FBS and 1% antibiotic/antimycotic solution at 37 °C in an atmosphere of 5% CO_2_. Before performing the seeding, the confluent cells, at passages 19–22, were detached from the culture flask by using TrypLE Express. The L929 cell line was seeded at a density of 1 × 10^4^ cells/well in an adherent 24-well culture plate and incubated for 24 h at 37 °C in a humidified atmosphere with 5% CO_2_. The culture medium was removed, and the same volume (1 mL) of different *S. officinalis* extracts at different concentrations (Section 2.7) was added. The L929 cell line in culture was incubated with the extracts for 24, 48, and 72 h, and the metabolic activity, DNA quantification, total protein content, and morphology of cells were evaluated for each time point. The negative control comprised the culture of the L929 cell line in medium (0 μg/mL) for each respective extract.

#### 2.8.2. Metabolic Activity

The metabolic activity of the L929 cell line was determined by the dehydrogenase activity of cells using the MTS assay, according to manufacturer’s instructions. After 24, 48, and 72 h of culture, the culture medium was removed, and the L929 cell line was gently washed twice with sterilized PBS. Serum-free culture medium without phenol red and MTS reagent were added to each well at a ratio of 5:1. MTS reagent was used as blank. Cells were incubated at 37 °C for 3 h in a humidified atmosphere containing 5% CO_2_. Thereafter, the absorbance of the MTS reaction medium from each sample was recorded in triplicate at 490 nm in a microplate reader. The results are expressed in percentage with respect to the control.

#### 2.8.3. DNA Quantification

Cell proliferation was determined using a fluorimetric dsDNA quantification kit, performed according to the manufacturer’s instructions. After 24, 48, and 72 h of culture, the culture medium was removed, and the L929 cell line was gently washed with sterilized PBS. Then, 1 mL of ultrapure water was added to each well. After 30 min, the samples were collected, transferred into Eppendorf tubes, and frozen at −80 °C. The thawed samples were sonicated (15 min), and 28.7 μL of the sample or standard (0 to 2 µg/mL) was added in triplicate to the white opaque 96-well plate, followed by PicoGreen solution (71.3 μL) and Tris-EDTA (TE) buffer (100 μL). The plate was incubated for 10 min in the dark, and the fluorescence of each sample was measured in a microplate reader (EX = 485 nm and EM = 528 nm). The DNA concentration (µg/mL) of each sample was calculated using the standard curve relating to the DNA concentration and the fluorescence intensity. The results are expressed in relative DNA concentration of the control.

#### 2.8.4. Total Protein Content

A micro bicinchoninic acid (BCA) protein assay kit was used for the quantification of total protein content, according to the manufacturer’s instructions. Samples were collected and prepared for assaying as described in DNA quantification. Then, 150 µL of samples and bovine serum albumin (BSA) standards (0 to 40 µg/mL), in triplicate, were mixed with the working reagent (150 µL) in a 96-well plate and incubated at 37 °C for 2 h in the dark. After that, the absorbance was read at 562 nm using a microplate reader. The total protein content (µg/mL) of each sample was calculated using the standard curve relating to the BSA concentration and the absorbance intensity. The results are expressed in relative total protein content of the control.

#### 2.8.5. Cell Morphology

A high-resolution field emission scanning electron microscope (HR-SEM, Auriga Compact, ZEISS) was used to analyze the morphology of the L929 cell line. After 24, 48, and 72 h of culture, the medium was removed, and the L929 cell line was rinsed with sterile PBS. To fix the cells to the bottom of the plate, 2.5% glutaraldehyde in PBS solution was added, and the plates were kept at 4 °C. The samples were dehydrated with increasing concentrations of ethanol (10%, 20%, 30%, 40%, 50%, 60%, 70%, 80%, 90%, and 100%) and dried overnight at RT. Afterwards, the bottom of the well was melted with a soldering-iron and placed in the stub. The samples were then sputter-coated (EM ACE600, Leica Mikrosysteme GmbH, Wien, Austria) with gold–palladium, and the micrographs were recorded at 5 kV with magnifications of 200× and 1000×.

### 2.9. Proinflammatory Activity Evaluation

Proinflammatory activity of *S. officinalis* extracts was evaluated using a human peripheral blood monocyte cell line (THP-1), obtained from American Type Culture Collection (ATCC^®^ TIB-202^TM^), according to a modified procedure described elsewhere [52,53]. The THP-1 cell line, at passages 9–14, was cultured in RPMI medium supplemented with 10% FBS and 1% antibiotic/antimycotic solution at 37 °C in an atmosphere of 5% CO_2_. The cells were seeded at a density of 5 × 10^5^ cells in an adherent 24-well culture plate. For the induction of THP-1 cell differentiation, the RPMI medium containing 100 nM phorbol 12-myristate 13-acetate (PMA) was added and incubated for 24 h. After this period of time, the nonattached cells were removed by aspiration, and the adherent cells were washed twice with warm RPMI medium. To ensure the reversion of monocyte to a resting macrophage phenotype, the cells were incubated for an additional 48 h in RPMI without PMA. Afterwards, the medium was changed, and each *S. officinalis* extracts at different concentrations (Section 2.7) were added to the nonstimulated macrophages. After a period of incubation of 24 h, the culture medium was harvested (the triplicates were mixed and homogenized) and stored aliquoted at −80 °C until cytokine quantification. The cells were washed with warm sterile PBS, and the metabolic activity and DNA quantification were performed. The cell morphology was analyzed before collecting the medium under an inverted microscope (Axio Vert.A1, Carl Zeiss Microscopy GmbH, Göttingen, Germany).

#### Metabolic Activity

The metabolic activity of nonstimulated macrophages incubated with *S. officinalis* extracts was determined by the reduction of the resazurin (blue) to resorufin (pink) by living macrophages using the alamarBlue assay. RPMI medium containing 10% alamarBlue was added to each well. A blank was also made (10% alamarBlue without cells). The cells were incubated at 37 °C for 4 h in a humidified atmosphere containing 5% CO_2_. Thereafter, the absorbance of the alamarBlue reduction from each sample was recorded in triplicate at 600 and 570 nm on a microplate reader. The results are expressed in percentage related to the control.

### 2.10. Anti-Inflammatory Activity Evaluation

The THP-1 cell line was seeded and cultured, as previously described (Section 2.9). After the total reversion of monocyte to macrophage phenotype, macrophages were stimulated with 100 ng/mL of LPS in fresh medium for 2 h to provide an inflammatory stimulus. *S. officinalis* extracts at different concentrations (Section 2.7) were then added to the LPS-stimulated macrophages and incubated for 22 h. Afterwards, the culture medium was harvested and stored as previously described. The cells were then washed with warm sterile PBS, and the metabolic activity, DNA quantification, and morphology were assessed, as previously described. LPS-stimulated macrophages cultured without extracts (only with culture medium, 0 μg/mL) were used as a positive control to stimulate IL-6 production. Dexamethasone, diclofenac, salicylic acid, and celecoxib at 10 µM, dissolved in ethanol, were used as positive controls of cytokine production inhibition. The negative control was cells without LPS stimulation.

#### IL-6 and TNF-α Quantification

The amount of IL-6 and TNF-α was assayed using an ELISA kit, according to the manufacturer’s instructions. The obtained values were normalized by the respective DNA concentration. The results are expressed in percentage related to the positive control.

### 2.11. Statistical Analysis

The results were obtained as 3 independent experiments with a minimum of 3 replicates for each condition and are expressed as mean ± standard deviation (SD). Statistical analyses were performed using GraphPad Prism 6.0 software. Analysis of variance (ANOVA) and Tukey’s multiple comparisons test were used for yield extraction, total phenolic and flavonoid content, and antioxidant activity. Analysis of variance (ANOVA) and Dunnett’s multiple comparison method were used for cell assays. Differences between experimental groups were considered significant with a confidence interval of 99%, whenever *p* < 0.01.

## 3. Results

### 3.1. Extraction Yield

**Figure 1:**
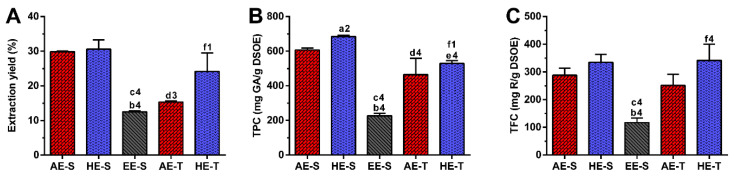
Extracts yield (**A**), total phenolic (TPC) (**B**), and flavonoid (TFC) (**C**) contents of *Salvia officinalis* extracts. Statistically significant differences are 1 (*p* < 0.0257), 2 (*p* < 0.0037), 3 (*p* < 0.0004), and 4 (*p* < 0.0001) in comparison with a (AE-S vs. HE-S), b (AE-S vs. EE-S), c (HE-S vs. EE-S), d (AE-S vs. AE-T), e (HE-S vs. HE-T), and f (AE-T vs. HE-T). GA: gallic acid; R: rutin; DSOE: dry *Salvia officinalis* extract; AE: aqueous extracts; HE: hydroethanolic extracts; EE: ethanolic extracts; S: soxhlet extraction; T: traditional extraction.

The extraction yield obtained for each extraction method is illustrated in Figure 1A. In the soxhlet extraction, similar extraction yields were obtained using a hydroethanolic solution (50:50) or water, 30.6 ± 2.7% and 29.9 ± 0.2%, respectively. The extraction with ethanol led to a significantly lower extraction yield (12.2 ± 0.3%) in comparison with the previous solvents. In the traditional extraction, the extraction yield was significantly higher using the hydroethanolic solution (24.2 ± 5.3%), followed by water (15.3 ± 0.3%). Comparing both techniques, significant differences were observed only for AE, where AE-S promoted a higher extraction yield than AE-T. Analyzing all the *S. officinalis* extracts, HE-S had a similar yield to AE-S, followed by HE-T, AE-T, and EE-S.

### 3.2. TPC and TFC

The TPC and TPF of *S. officinalis* extracts are presented in Figure 1B,C, respectively. The TPC was significantly higher in HE-S (685.2 ± 6.6 mg GA/g DSOE), followed by AE-S (606.3 ± 11.5 mg GA/g DSOE) and EE-S (227.2 ± 13.2 mg GA/g DSOE). In traditional extraction, HE-T significantly recovered a higher phenol content (528.8 ± 17.1 mg GA/g DSOE) compared with AE-T (464.5 ± 94.0 mg GA/g DSOE). Moreover, a significantly higher TPC was observed in AE-S and HE-S compared with AE-T and HE-T. Analyzing all the *S. officinalis* extracts, HE-S presented a higher amount of TPC, followed by AE-S, HE-T, AE-T, and EE-S.

Similar amounts of flavonoids were found in soxhlet extraction performed with HE-S (334.7 ± 29.3 mg R/g DSOE) and AE-S (287.9 ± 25.2 mg R/g DSOE), while EE-S extracted significantly lower TFC (117.0 ± 16.7 mg R/g DSOE). HE-T recovered a significantly higher flavonoid content (342.0 ± 57.7 mg R/g DSOE) compared with AE-T (251.6 ± 40.1 mg R/g DSOE). No statistically significant differences in TFC were found for both extraction techniques, being the values similar. Analyzing all the *S. officinalis* extracts, HE-S and HE-T presented a higher amount of TFC, followed by AE-S, AE-T, and EE-S.

### 3.3. TLC Analysis of S. officinalis Extracts

**Figure 2:**
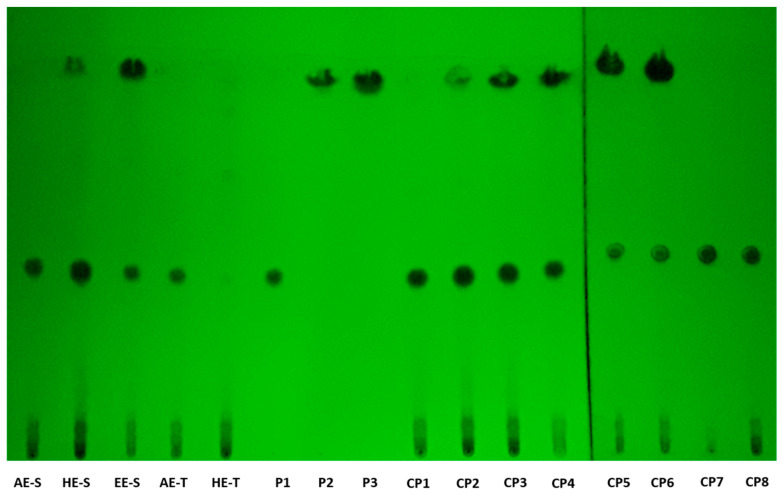
Thin-layer chromatography (TLC) chromatogram of *Salvia officinalis* extracts and standards. AE: aqueous extracts; HE: hydroethanolic extracts; EE: ethanolic extracts; S: soxhlet extraction; T: traditional extraction; P1: rosmarinic acid, P2: carnosol; P3: carnosic acid; CP1: AE-S + P1; CP2: HE-S + P1; CP3: HE-S + P2; CP4: EE-S + P1; CP5: EE-S + P2; CP6: EE-S + P3; CP7: AE-T + P1; CP8: HE-T + P1.

TCL analysis of the five *S. officinalis* extracts revealed similar chromatographic profiles (Figure 2). Several standards were tested; however, at most, only three different spots appeared in the *S. officinalis* extracts. Rosmarinic acid (R_F_ = 0.443 ± 0.007) appeared in all extracts. Carnosol (R_F_ = 0.935 ± 0.012) and carnosic acid (R_F_ = 0.948 ± 0.017), with a specific shape, could be observed in HE-S and EE-S. The samples and standards together corroborated these observations.

### 3.4. Antiradical Activity of S. officinalis Extracts against DPPH^●^ and ABTS^●+^

**Figure 3:**
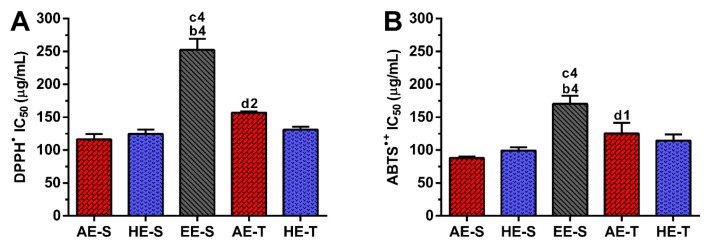
Half-maximal inhibitory concentration (IC_50_, μg/mL) of the different *Salvia officinalis* extracts against DPPH^●^ (**A**) and ABTS^●+^ (**B**). Statistically significant differences are 1 (*p* < 0.0128), 3 (*p* < 0.0081), and 4 (*p* < 0.0001) in comparison with b (AE-S vs. EE-S), c (HE-S vs. EE-S), and d (AE-S vs. AE-T). AE: aqueous extracts; HE: hydroethanolic extracts; EE: ethanolic extracts; S: soxhlet extraction; T: traditional extraction.

A screening of the antiradical activity of *S. officinalis* extracts was firstly evaluated against the most common radicals, namely DPPH^●^ and ABTS^●+^, due to their simple measurement. The IC_50_ (μg/mL) of each *S. officinalis* extract is presented in Figure 3. All *S. officinalis* extracts showed antiradical activity against DPPH^●^ (Figure S1) and ABTS^●+^ (Figure S2) in a concentration-dependent manner.

In the soxhlet extraction, the IC_50_ for DPPH^●^ of AE-S (116.5 ± 7.9 μg/mL) and HE-S (124.7 ± 6.6 μg/mL) did not present significant differences, but both were significantly lower in comparison with EE-S (252.3 ± 17.0 μg/mL) (Figure 3A). In the traditional extraction, AE-T (157.0 ± 1.9 μg/mL) and HE-T (131.3 ± 4.1 μg/mL) also did not show significant differences in the IC_50_ values. Comparing both techniques, significant differences were observed only for AE as AE-S showed higher antiradical activity than AE-T. No differences were found for HE, being the values similar. Analyzing all the *S. officinalis* extracts, AE-S presented higher DPPH^●^ scavenging activity, followed by HE-S, HE-T, AE-T, and EE-S.

AE-S (88.2 ± 2.3 μg/mL) and HE-S (99.3 ± 4.1 μg/mL) showed similar IC_50_ against ABTS^●+^, but significantly higher activity in comparison with EE-S (170.6 ± 12.1 μg/mL) (Figure 3B). In the traditional extraction, HE-T (114.5 ± 9.6 μg/mL) had a comparable IC_50_ to the AE-T (125.2 ± 16.1 μg/mL). Comparing both extraction techniques, only AE showed significant differences, presenting the soxhlet extraction extracts the lowest IC_50_. Analyzing all the *S. officinalis* extracts, AE-S presented higher ABTS^●+^ scavenging activity, followed by HE-S, HE-T, AE-T, and EE-S.

### 3.5. Antioxidant Activity against ROO^●^

**Figure 4:**
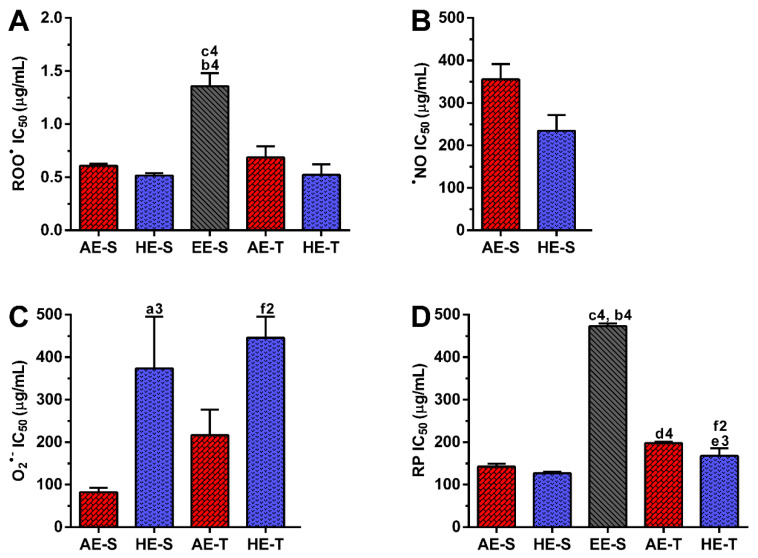
Half-maximal inhibitory concentration (IC_50_, μg/mL) of the different *Salvia officinalis* extracts against ROO^●^ (**A**), ^●^NO (**B**), and O_2_^●−^ (**C**), as well as their reducing power (RP) (**D**). Statistically significant differences are 1 (*p* < 0.0105), 2 (*p* < 0.0032), 3 (*p* < 0.0002), 4 (*p* < 0.0001) in comparison with a (AE-S vs. HE-S), b (AE-S vs. EE-S), c (HE-S vs. EE-S), d (AE-S vs. AE-T), e (HE-S vs. HE-T), and f (AE-T vs. HE-T). AE: aqueous extracts; HE: hydroethanolic extracts; EE: ethanolic extracts; S: soxhlet extraction; T: traditional extraction.

All *S. officinalis* extracts showed scavenging activity for ROO^●^ in a concentration-dependent manner (Figure S3). The antioxidant activity against ROO^●^ (Figure 4A) was similar for HE-S (0.52 ± 0.02 μg/mL) and AE-S (0.61 ± 0.02 μg/mL) and significantly higher compared to EE-S (1.35 ± 0.12 μg/mL). The same tendency occurred in the traditional extraction, where IC_50_ values from AE-T (0.69 ± 0.12 μg/mL) and HE-T (0.52 ± 0.10 μg/mL) did not present statistical differences. Extracts obtained with a soxhlet or by the traditional method presented a similar antioxidant activity against ROO^●^. Analyzing all the *S. officinalis* extracts, HE-S and HE-T presented higher antioxidant activity against ROO^●^, followed by AE-S, AE-T, and EE-S.

### 3.6. Antioxidant Activity against ^●^NO

The different types of *S. officinalis* extracts presented the ability to scavenge ^●^NO in a concentration-dependent manner (Figure S4). In this assay, EE-S, AE-T, and HE-T did not present the ability to reduce 50% of the ^●^NO amount. HE-S (234.6 ± 36.9 μg/mL) showed to have more antioxidant effects against ^●^NO in comparison with AE-S (355.6 ± 35.9 μg/mL) (Figure 4B). At a concentration of 500 μg/mL, HE-S neutralized 56.26 ± 5.5% of nitrite, followed by AE-S (50.93 ± 2.7%), EE-S (43.23 ± 3.8%), HE-T (41.59 ± 1.0%), and AE-T (38.54 ± 3.0%).

### 3.7. Antioxidant Activity against O_2_^●−^

The different *S. officinalis* extracts showed scavenging activity for O_2_^●−^ in a concentration-dependent manner (Figure S5). The IC_50_ was significantly higher for AE-S (82.3 ± 10.0 μg/mL) compared to HE-S (378.4 ± 128.8 μg/mL) (Figure 4C) but was not possible to calculate for EE-S. The same behavior was observed in the traditional extraction, where the antioxidant activity against O_2_^●−^ was significantly higher for AE-T (216.3 ± 60.0 μg/mL) in comparison with HE-T (450.7 ± 56.8 μg/mL). There are no significant differences between the different extraction techniques. At a concentration of 500 μg/mL, AE-T neutralized 70.3 ± 5.5% of O_2_^●−^, followed by AE-S (68.9 ± 4.1%), HE-T (61.5 ± 5.9%), HE-S (53.3 ± 12.7%), and EE-S (25.8 ± 8.6%).

### 3.8. RP Scavenging Activity

All *S. officinalis* extracts showed RP in a concentration-dependent manner (Figure S6). In the soxhlet extraction, no differences were observed in the IC_50_ values for RP between HE-S (126.9 ± 3.9 μg/mL) and AE-S (142.3 ± 6.7 μg/mL) (Figure 4D). However, both extracts presented significant differences in comparison with EE-S (473.0 ± 6.1 μg/mL). Traditional extraction provided HE-T (167.5 ± 18.0 μg/mL) with lower reducing power in comparison with AE-T (197.4 ± 3.4 μg/mL). Significant differences were observed in AE and HE obtained from soxhlet extraction or traditional extraction. Analyzing all the *S. officinalis* extracts, HE-S presented lower RP, followed by AE-S, HE-T, AE-T, and EE-S.

### 3.9. Cytotoxicity of S. officinalis Extracts

#### 3.9.1. L929 Cell Line

**Figure 5:**
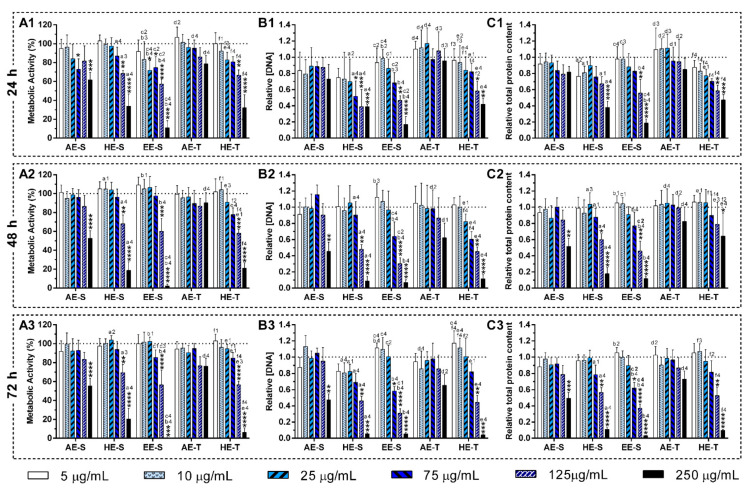
Metabolic activity (**A**), relative DNA concentration (**B**) and relative total protein content (**C**) of the L929 cell line in the presence of different concentrations of the *Salvia officinalis* extracts over 24 h (A1, B1, and C1), 48 h (A2, B2, and C2), and 72 h (A3, C3 and B3) of culture. The dotted line represents the nontreated condition (0 μg/mL) for each assay. Statistically significant differences are * (*p* < 0.0466), ** (*p* < 0.0093), *** (*p* < 0.0010), and **** (*p* < 0.0001) in comparison with the negative control (0 mg/mL) for each different *Salvia officinalis* extracts, and 1 (*p* < 0.0496), 2 (*p* < 0.0085), 3 (*p* < 0.0009), and 4 (*p* < 0.0001) in comparison with a (AE-S vs. HE-S), b (AE-S vs. EE-S), c (HE-S vs. EE-S), d (AE-S vs. AE-T), e (HE-S vs. HE-T), and f (AE-T vs. HE-T). AE: aqueous extracts; HE: hydroethanolic extracts; EE: ethanolic extracts; S: soxhlet extraction; T: traditional extraction.

The cytotoxicity of the different *S. officinalis* extracts was firstly performed with a mouse adipose fibroblasts cell line (L929). Figure 5 shows the metabolic activity, cell proliferation, and total protein content of the fibroblasts in the presence or absence of *S. officinalis* extracts for 72 h of culture. AE-S did not compromise the metabolic activity of fibroblasts for concentrations lower than 125 μg/mL, while HE-S and EE-S affected the metabolic activity for concentrations higher than 75 μg/mL for 72 h of culture (Figure 5A) in comparison with control (0 μg/mL). Additionally, EE-S significantly affected the metabolic activity of fibroblasts for concentrations higher than 25 μg/mL in the first 24 h of culture. However, the cells were able to recover their metabolic activity in the next 24 h. Fibroblasts were able to proliferate (Figure 5B) and synthesize protein (Figure 5C) for 72 h of culture when incubated with the *S. officinalis* extracts for the above-mentioned concentrations. In the traditional extraction, AE-T in all tested concentrations did not affect the metabolic activity (Figure 5A), even the cell proliferation (Figure 5B) and protein content (Figure 5C), of fibroblasts in all tested concentrations for 72 h of cell culture. HE-T significantly influenced the metabolic activity (Figure 5A), as well as the cell proliferation (Figure 5B) and protein content (Figure 5C), for concentrations higher than 125 μg/mL for 72 h of culture.

Cell morphology was not affected at cytocompatible concentrations of *S. officinalis* extracts during the culture time considered, according to HR-SEM micrographs (Figures S7–S12). All the samples presented a typical elongated morphology and pronounced filopodia similar to the control (0 μg/mL, Figure S7), and a higher number of cells was observed over time. Only EE-S (Figure S10) and HE-T (Figure S12) at 125 and 250 μg/mL, respectively, drastically changed the fibroblast’s morphology, whereby a lower number of fibroblasts were observed and having a more rounded-like shape.

AE-S were significantly more cytotoxic in comparison with AE-T. Contrarily, HE-S was more cytocompatible in comparison with HE-T. Analyzing all the *S. officinalis* extracts, the L929 cell line was more cytocompatible with AE-T, followed by AE-S, HE-S, HE-T, and EE-S.

#### 3.9.2. Non- and LPS-Stimulated Macrophages

**Figure 6:**
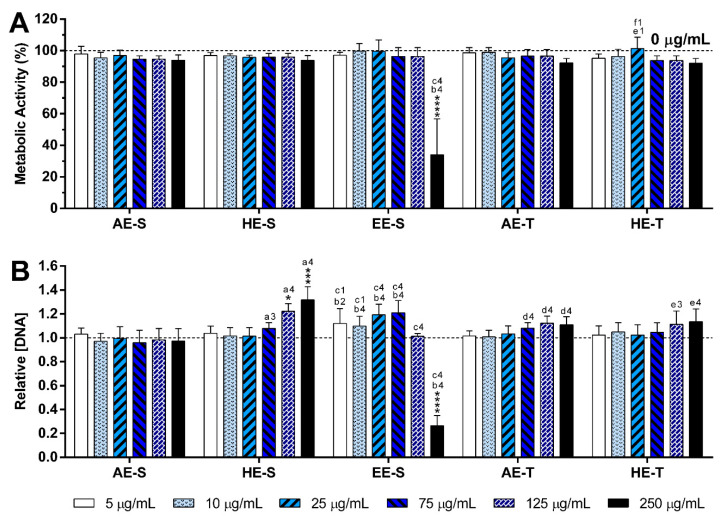
Metabolic activity (**A**) and relative DNA concentration (**B**) of nonstimulated macrophages cultured in the presence of different concentrations of the *Salvia officinalis* extracts for 24 h of culture at 37 °C. The dotted line represents the nontreated condition (0 μg/mL) for each assay. Statistically significant differences are * (*p* < 0.0384), *** (*p* < 0.00109) and **** (*p* < 0.0001) in comparison to the negative control (0 μg/mL) for each different tested extract, and 1 (*p* < 0.0251), 2 (*p* < 0.095), 3 (*p* < 0.0005), and 4 (*p* < 0.0001) in comparison with a (AE-S vs. HE-S), b (AE-S vs. EE-S), c (HE-S vs. EE-S), d (AE-S vs. AE-T), e (HE-S vs. HE-T), and f (AE-T vs. HE-T). AE: aqueous extracts; HE: hydroethanolic extracts; EE: ethanolic extracts; S: soxhlet extraction; T: traditional extraction.

**Figure 7:**
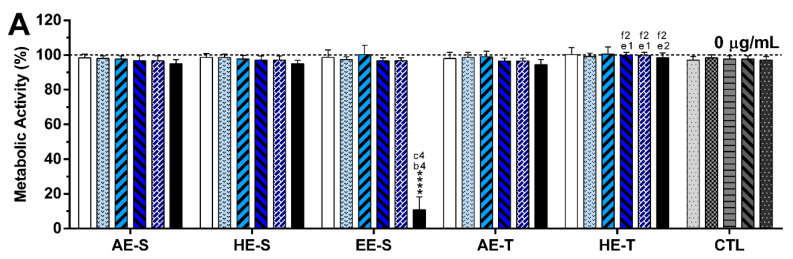

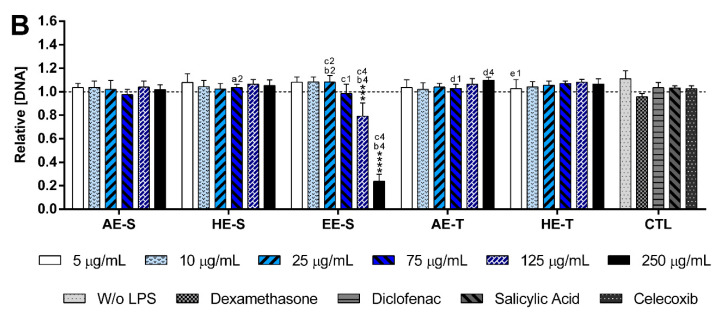
Metabolic activity (**A**) and relative DNA concentration (**B**) of LPS-stimulated macrophages cultured in the presence of different concentrations of the *Salvia officinalis* extracts and clinically used anti-inflammatory drugs (dexamethasone, diclofenac, salicylic acid and celecoxib, 10 μM) for 24 h of culture at 37 °C. The dotted line represents the nontreated condition (0 μg/mL) for each assay. Statistically significant differences are *** (*p* < 0.0006) and **** (*p* < 0.0001) in comparison to the negative control (0 μg/mL) for each different tested extract, and 1 (*p* < 0.0376), 2 (*p* < 0.0090), and 4 (*p* < 0.0001) in comparison with a (AE-S vs. HE-S), b (AE-S vs. EE-S), c (HE-S vs. EE-S), d (AE-S vs. AE-T), e (HE-S vs. HE-T), and f (AE-T vs. HE-T). AE: aqueous extracts; HE: hydroethanolic extracts; EE: ethanolic extracts; S: soxhlet extraction; T: traditional extraction; CTL: control.

The metabolic activity and relative DNA concentration obtained for nonstimulated macrophages (Figure 6) and LPS-stimulated macrophages (Figure 7) in the absence or presence of the *S. officinalis* extracts at different concentrations are similar, except for the highest tested concentration (250 μg/mL) for EE-S. The DNA of macrophages was preserved in the presence of the different *S. officinalis* extracts (Figure 6B) and under an inflammatory scenario (Figure 7B). Only the EE-S in the highest concentration (250 μg/mL) significantly reduced the DNA concentration of nonstimulated macrophages, which was even more pronounced in LPS-stimulated macrophages, which is in agreement with metabolic activity results. No significant differences were observed between extraction techniques.

Optical micrographs of nonstimulated macrophages (Figures S14 and S15) and LPS-stimulated macrophages (Figures S16 and S17) confirmed that the cell morphology was not affected by the different *S. officinalis* extracts at different concentrations. All the tested conditions showed a macrophage-like-phenotype similar to the negative control (0 μg/mL, Figure S13) in all tested conditions. However, the morphology of macrophages was only drastically affected by the presence of EE-S for concentrations higher than 125 μg/mL (Figures S14 and S16). These results are in agreement with previous assays.

### 3.10. Pro- and Anti-Inflammatory Activity of S. officinalis Extracts

#### 3.10.1. Nonstimulated Macrophages

The proinflammatory activity of cytocompatible *S. officinalis* extracts was evaluated by assessing the levels of IL-6 and TNF-α produced by non-LPS-stimulated macrophages in the cell culture medium. Nonstimulated macrophages without treatment (0 μg/mL, negative control) did not produce measurable amounts of IL-6 but produced small amounts of TNF-α (2.8 ± 2.0 pg/mL). Indeed, the production of basal levels of proinflammatory cytokines has been reported for macrophages [54]. When macrophages were incubated with *S. officinalis* extracts at different concentrations, a significant increase of the IL-6 and TNF-α levels was not observed in the culture medium.

#### 3.10.2. LPS-Stimulated Macrophages

**Figure 8:**
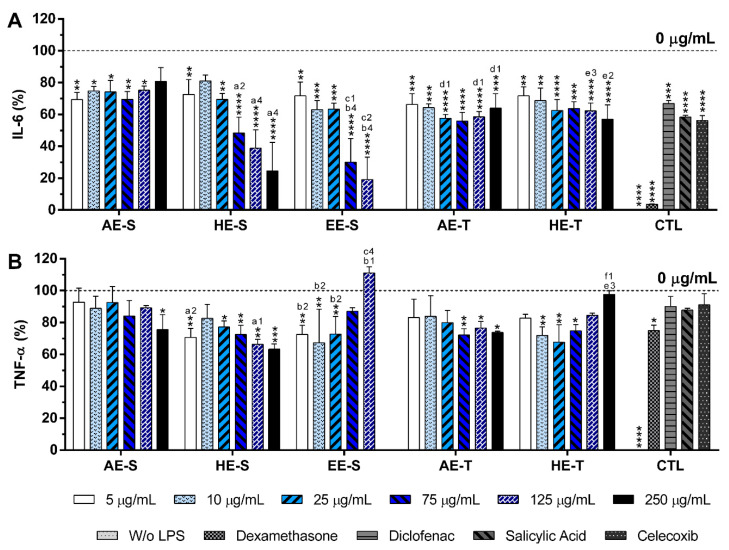
IL-6 (**A**) and TNF-α (**B**) percentages of LPS-stimulated macrophages cultured in the presence of different concentrations of the *Salvia officinalis* extracts and clinically used anti-inflammatory drugs (dexamethasone, diclofenac, salicylic acid, and celecoxib, 10 μM) for 24 h of culture at 37 °C. The dotted line represents the nontreated condition (0 μg/mL) for each assay. Statistically significant differences are * (*p* < 0.0474), ** (*p* < 0.0092), *** (*p* < 0.0008), **** (*p* < 0.0001) in comparison to the positive control (0 μg/mL) for each different tested extracted, and 1 (*p* < 0.0381), 2 (*p* < 0.0085), 3 (*p* < 0.0009), and 4 (*p* < 0.0001) in comparison with a (AE-S vs. HE-S), b (AE-S vs. EE-S), c (HE-S vs. EE-S), d (AE-S vs. AE-T), e (HE-S vs. HE-T), and f (AE-T vs. HE-T). AE: aqueous extracts; HE: hydroethanolic extracts; EE: ethanolic extracts; S: soxhlet extraction; T: traditional extraction; CTL: control.

The stimulation of macrophages with LPS (100 ng/mL) led to a significant production of IL-6 and TNF-α (Figure 8), which is not observed in nonstimulated macrophages, as previously verified.

Dexamethasone, diclofenac, salicylic acid, or celecoxib at 10 μM led to a statistically significant reduction of IL-6 production by 96.4 ± 0.2%, 33.1 ± 1.8%, 41.6 ± 1.2%, and 43.7 ± 3.1%, respectively, being the first and most efficient control. In the presence of all *S. officinalis* extracts, IL-6 production was also statistically significantly reduced. *S. officinalis* extracts obtained from soxhlet extraction were more efficient in the reduction of IL-6 production than the extracts obtained from traditional extraction. Treatment with HE-S and EE-S drastically decreased the IL-6 production in a concentration-dependent manner. HE-S and EE-S, at a cytocompatible concentration (75 μg/mL), decreased the IL-6 production by 51.6 ± 10.0% and 70.0 ± 14.9%, respectively. In the case of the AE-S, AE-T, and HE-T, the influence of the extract concentration in the reduction of the IL-6 production was observed. A small concentration (75 μg/mL) of AE-S and AE-T were required to inhibit 30.5 ± 5.0 and 44.2 ± 5.5% of IL-6 production, respectively. HE-T could reduce 37.7 ± 4.9% of IL-6 production with a concentration of 125 μg/mL. Analyzing all the *S. officinalis* extracts, the reduction of IL-6 production was more pronounced with EE-S, followed by HE-S, HE-T, AE-T, and AE-S.

Dexamethasone (10 μM) significantly reduced TNF-α production by a percentage of 25.0 ± 3.3%. Under this in vitro inflammatory model, a reduction of 9.9 ± 6.3%, 12.2 ± 1.2%, and 8.9 ± 6.9% of the TNF-α amount was obtained for diclofenac, salicylic acid, and celecoxib at 10 μM, respectively, but no significant differences were observed (Figure 8B). Conversely, *S. officinalis* extracts were able to significantly reduce the TNF-α amount. The efficacy of extracts to reduce TNF-α production was not influenced by the extraction technique. AE-T were more efficient in the reduction of this proinflammatory cytokine in comparison with AE-S, while HE-S showed more anti-inflammatory activity than HE-T. HE-S, at 250 μg/mL, led to the higher reduction of the TNF-α amount (36.7 ± 3.4%), even if compared with all the tested controls. A similar reduction of this cytokine was observed for EE-S (32.7 ± 21.1% at 10 μg/mL) and HE-T (32.2 ± 10.8%, at 25 μg/mL) at lower concentrations AE-TE (75 μg/mL) and AE-S (250 μg/mL) also led to a significant reduction of TNF-α production by 27.8 ± 3.9% and 24.4 ± 9.3%, respectively.

## 4. Discussion

Powerful benefits of *S. officinalis* were reported already by the Romans; its leaves were considered as a medicine to enhance health and treat ailments, especially in inflammatory disorders [32]. Inspired by this, *S. officinalis* leaves were used to obtain new extracts with the ability to treat inflammatory diseases. In the literature, the most frequently used extraction solvents for *S. officinalis* are water, methanol, ethanol, acetone, hexane, and ethyl acetate, resulting in extracts enriched in phenols (carnosic acid, carnosol, methyl carnosate, rosmarinic acid, rosmanol, rosmadial, epirosmanol, caffeic acid, ferulic acid), flavonoids (apigenin, apigenin 7-*O*-glucoside, luteolin, luteolin-7-*O*-glucoside, luteolin 7-*O*-glucuronide), and essential oils (1,8-cineole, α/β-thujone, camphor, camphene, borneol, bornyl acetate, β-pinene, manool, viridiflorol [55,56,57,58,59,60,61,62,63,64]. In this work, the process of extraction was first and exactly carried as it has been traditionally performed for years. In this sense, AE-T and HE-T were obtained by infusion and maceration, respectively. A soxhlet apparatus was then used to obtain these two types of extracts (AE-S and HE-S), as well as EE-S. Indeed, the efficiency of extraction can be affected by several parameters, such as the chemical nature of the bioactive compounds, extraction method, weight and size of the plant samples, as well as solvent, pH, time, and temperature of extraction [65]. Therefore, as expected, in this work, the extraction yield was strongly influenced by the extraction technique and the solvent used (Figure 1A). Soxhlet extraction provided higher efficiency (≈50%) than traditional extraction in the recovery of hydrophilic compounds from *S. officinalis*, since the combination of water with heating for longer periods of time promoted more efficient cell disruption, increasing the extraction capacity [66]. In maceration, the contact between the plant sample and solvent was maintained for five days, which promoted a similar amount of extractable hydroethanolic compounds in comparison with the soxhlet extraction.

Regarding TPC, *S. officinalis* is the species of Salvia that presents the highest content of phenolic compounds [67]. The amount of phenolic compounds (Figure 1B) was significantly higher in the soxhlet extraction than in the traditional extraction, whereas the amount of flavonoid compounds (Figure 1C) was higher in hydroethanolic extracts compared to the other types of extracts. These results emphasize the important role of the temperature and polarity of the solvent in both phenol and flavonoid extraction, respectively. Although the extraction yield did not present statistically significant differences between AE-S and HE-S and between HE-S and HE-T, their TPC was statistically different. This observation emphasizes that other compounds behind phenols (and flavonoids), such as proteins, carbohydrates, and polysaccharides, have been extracted from *S. officinalis* leaves, contributing, therefore, to enhancing its extraction yield. There are different studies reporting the TPC of several *S. officinalis* or other plant species extracts, prepared with different extraction techniques [54,57,63,68,69,70]. The TPC and TFC of the *S. officinalis* extracts obtained in this study were significantly higher than the values obtained for similar ethyl acetate extracts produced by Kontogianni et al. [40]. As both extracts were prepared with a soxhlet, the selection of solvents may be the main responsible for these differences in phenols and flavonoids amounts.

According to TLC analysis, rosmarinic acid, carnosol, and carnosic acid are present in *S. officinalis* extracts (Figure 2). Rosmarinic acid was presented in all extracts, meaning that the solvent and technique of extraction did not affect its recovery. Rosmarinic acid was also detected by TLC in AE and HE obtained by the traditional method [71,72]. To extract carnosol and carnosic acid (HE-S and EE-E), high temperatures and the presence of ethanol in the solvent of extraction were required.

Phenolic and flavonoid compounds, such as rosmarinic acid, carnosol, and carnosic acid present antioxidant activity [58,73,74]. Therefore, the antioxidant ability of *S. officinalis* extracts against DPPH^●^ and ABTS^●+^ (Figure 3), as well as ROO^●^, ^●^NO, and O_2_^●−^ and reducing power (Figure 4), were all evaluated. Overall, depending on the assayed extracts and reactive species, all *S. officinalis* extracts exhibited significant antioxidant activity (Figures S1–S5). They were also able to reduce the ferric form (Fe^3+^) into ferrous (Fe^2+^) in a concentration-dependent manner (Figure S6). This demonstrates the phytochemical compounds’ ability to donate a hydrogen atom and neutralize the radicals [42,43,44,45,47,48]. The differences in the activities among the different extracts could be related to the extracts’ composition and the relative amount of certain compounds. Lu et al. demonstrated that *S. officinalis* phenols are more powerful in scavenging DPPH^•^ than flavonoids, which have only weak to moderate activities [74]. Rosmarinic acid was detected in all *S. officinalis* extracts, and carnosic acid is present in HE-S and EE-S. Those compounds have two catechol groups in the aromatic ring of the phenolic skeleton, presenting a strong antioxidant activity [57]. Other compounds, such as caffeoyl derivatives, with a chemical structure similar to rosmarinic acid, are also abundant in Lamiaceae species and can contribute to the antioxidant activity of the extracts [57]. Indeed, the antioxidant properties of single compounds within a group can vary remarkably so that the same levels of phenolic compounds do not correspond to the same antioxidant responses [75]. Moreover, some phenolic compounds cannot be quantified by the Folin–Ciocalteu method [75], whereby the radical scavenging activity of an extract cannot be deduced only by its TPC. In fact, the relationship between yield extraction, TPC, and its antioxidant activity is not always closely correlated [29]. For instance, methanol/water extracts obtained by stirring showed higher antiradical activity against DPPH^•^ than AE (decoction). However, the latter presented the highest concentration in phenolic and flavonoids compounds [56]. Moreover, HE produced in an enamel boiler by Kozics et al. demonstrated higher antiradical activity against DPPH^•^ and ABTS^●+^ than the *S. officinalis* extracts herein tested [76]. These differences can arise from the different extraction techniques and the experimental conditions used in the antiradical assay. A similar result was observed for extracts prepared in a soxhlet apparatus, but with an IC_50_ two times lower than the values obtained for the extracts herein studied [40]. In this case, the assay was performed in the same way, highlighting the importance of the extraction solvents in the biological activity of the extract. The authors started the extraction of leaves with hexane, followed by ethyl acetate. The hexane may remove compounds with no biological activity, while ethyl acetate may concentrate them. Interestingly, there are few studies reporting the antioxidant activity of *S. officinalis* extracts against ROO^●^, ^●^NO, and O_2_^●−^. A mixture of flowers, leaves, and stems of *S. officinalis* was extracted by decoction and defatted with n-hexane [77]. These extracts presented around two times more antioxidant activity against O_2_^●−^, while similar antioxidant activity against ^●^NO is observed. Again, these results show the importance of the solvent in the extraction of the biologically active compounds, as well as the interference of the different parts of plants.

Soxhlet extracts were more powerful scavengers for all the tested radicals (lower IC_50_) compared with their counterparts obtained in traditional extracts, which could be related to the higher TPC and TFC. However, the most pronounced antioxidant effect was observed for AE-S, since they have the lowest IC_50_ for all tested radicals. Moreover, these extracts present antioxidant activity against ^•^NO, which is not observed for AE-T. The antioxidant activity against O_2_^●−^ was also higher for these extracts in comparison with HE-T. Conversely, EE-S exhibited the lowest antioxidant activity. Similar to our results, an ethanolic extract of *Mentha rotundifolia* exhibited the lowest TPC; however, it was the most effective in the protection of lipid and protein peroxidation in the cell membrane of red blood cells than *Mentha pulegium* extract with higher TPC [54]. Therefore, during an inflammatory response, AE-S can neutralize the reactive molecules [2,3,10], terminate free-radical chain reactions (e.g., lipid peroxidation) by converting free radicals to more stable products [10], and ameliorate, e.g., the vascular permeability of the inflamed tissues, reducing the extravasation of immune cells [3]. Consequently, a cascade of reactions that lead to inflammatory and degenerative diseases, could be prevented. However, as *S. officinalis* extracts are also good electron donors, excess of antioxidant compounds in the presence of free metal ions, such as Fe or Cu, can also initiate and promote free radicals’ reactions and, consequently, an undesired pro-oxidative effect can be obtained [10,78]. Nonetheless, the oxidized forms of *S. officinalis* extracts may be relatively unreactive, and, consequently, do not cause cellular damage. In general, AE were more cytocompatible with the L929 cell line, whereas EE-S must have cytotoxic compounds for these fibroblasts (Figure 5A). Moreover, within the range of cytocompatible concentrations, fibroblasts were able to proliferate (Figure 5B) and synthesize proteins (Figure 5C) for 72 h of culture. Interestingly, some *S. officinalis* extracts were able to stimulate the mitogenic activity and protein synthesis, mainly AE, since higher amounts of DNA and protein concentration were observed in comparison with control (0 μg/mL). This effect can be related to the cytoprotective effect of *S. officinalis* extracts due to their antioxidant activity [54]. Furthermore, a fibroblast-like-phenotype was observed for *S. officinalis* extracts at cytocompatible concentrations (Figures S7–S12). These results are in agreement with the data of metabolic activity, relative DNA, and total protein concentrations obtained.

The immunomodulatory activity of *S. officinalis* extracts was assessed using macrophages since they are key players of the immune system [79]. To model an inflammatory scenario, macrophages were activated in vitro with LPS, which triggers a cascade of inflammatory pathways, including the induction of the production of proinflammatory cytokines (e.g., IL-6 and TNF-α) and ROS/RNS [80]. *S. officinalis* extracts were cytocompatible with both nonstimulated (Figure 6, Figures S14 and S15) and LPS-stimulated macrophages (Figure 7, Figures S16 and S17). Only EE-S in the highest tested concentration (250 μg/mL) significantly affected the macrophages’ metabolic activity, DNA concentration, and morphology, demonstrating its cytotoxicity if present in high amounts.

None of the *S. officinalis* extracts showed the ability to induce the production by nonstimulated macrophages of the proinflammatory cytokines IL-6 and TNF-α, suggesting that they lack immunostimulatory activity over macrophages. Conversely, our results indicated that all five *S. officinalis* extracts at cytocompatible concentrations efficiently reduced IL-6 and TNF-α production by LPS-stimulated macrophages (Figure 8). A comparable reduction of the IL-6 and TNF-α production was also observed in the oxidized low-density lipoproteins (ox-LDL) simulated macrophages (THP-1 cell line) in the presence of supercritical *S. officinalis* extracts [81]. The traditional extraction provided the AE with stronger anti-inflammatory activity in the reduction of IL-6 and TNF-α production. In fact, traditional extraction can retain the volatile components of *S. officinalis*, while they may be lost in a soxhlet extraction. These volatile compounds, such as camphor and borneol, reportedly have anti-inflammatory activity [34,37]. When the extracts were prepared using HE, the soxhlet originated stronger anti-inflammatory extracts, suggesting that this extraction technique recovered more compounds with the ability to reduce the IL-6 and TNF-α production, such as rosmarinic acid, carnosic acid, and carnosol [82,83,84]. Interestingly, EE-S was the most powerful anti-inflammatory extract that should be strongly related to the presence of rosmarinic acid, carnosol, and carnosic acid, followed by HE-S (presence of rosmarinic acid, carnosol, and carnosic acid) and AE-S (presence of rosmarinic acid). This result pointed out that solvent selection is extremely important to recover extracts with strong activity in reducing inflammatory cytokines production. Indeed, this highlights that the quality of the bioactive compounds that comprise the extract strongly influenced its biological activities.

A reduction of IL-6 production in a concentration-dependent manner was observed for both HE-S and EE-S, whereas the reduction of TNF-α in a concentration-dependent manner was only detected when macrophages were cultured in the presence of HE-S. A low amount of *S. officinalis* extracts (AE-S, AE-T, and HE-T, 5 μg/mL) was able to reduce IL-6 production. Moreover, at cytocompatible concentrations (75 μ/mL), the *S. officinalis* extracts were able to attenuate the TNF-α production by LPS-stimulated macrophages. Indeed, *S. officinalis* extracts led to a similar or higher decrease of the IL-6 and TNF-α amount than the well-known tested NSAIDs, namely diclofenac, salicylic acid, and celecoxib. Additionally, EE-S and HE-S showed similar anti-inflammatory activities of dexamethasone, a potent corticosteroid, but with important side effects. Thus, a formulation of *S. officinalis* extracts can be a promising therapeutic strategy for the reduction of the inflammatory process.

## 5. Conclusions

The present study showed that the selection of solvents and extraction techniques should be carefully considered since they influence the bioactivity and toxicity of the obtained extracts. AE-S obtained from *S. officinalis* leaves, enriched in phenols (e.g., rosmarinic acid) and flavonoids, showed to contain the best antioxidant mixture against dangerous free radicals. However, EE-S, with the lowest antioxidant activity, was the most promising extract in the reduction of IL-6 and TNF-α production by human macrophages, suggesting that rosmarinic acid, carnosol, and carnosic acid may be responsible for this activity. These results proved that *S. officinalis* leaves contain valuable substances. Therefore, a formulation combining, for instance, AE-S and EE-S would be a strong and promising antioxidant and anti-inflammatory mixture to counteract chronic inflammatory pathways, probably with low side effects. Therefore, *S. officinalis* extracts can be used as an effective and safe source of new drugs to treat chronic inflammatory diseases. Further in-depth studies are required to specifically determine which compounds present in *S. officinalis* extracts may really contribute to antioxidant and anti-inflammatory activities.

## Supplementary Materials

The following are available online at https://www.mdpi.com/2076-3921/9/11/1157/s1.
