# Supplementary material for: Antioxidant and Anti-Inflammatory Activities of Cytocompatible Salvia officinalis Extracts: A Comparison between Traditional and Soxhlet Extraction
Source: Antioxidants (Basel). 2020 Nov 20;9(11):1157. doi: 10.3390/antiox9111157 (PMC7699719; doi:10.3390/antiox9111157)
Supplement: Supplementary file 1 [file antioxidants-09-01157-s001.pdf]

antioxidants

Supplementary Materials

Antioxidant and anti-inflammatory activities of cytocompatible *Salvia officinalis* extracts: a comparison between traditional and soxhlet extraction

Sara F. Vieira [1,2], Helena Ferreira [1,2] and Nuno M. Neves [1,2,*]

[1] 3B's Research Group, I3BS – Research Institute on Biomaterials, Biodegradables and Biomimetics, University of Minho, Headquarters of the European Institute of Excellence on Tissue Engineering and Regenerative Medicine, AvePark, Parque de Ciência e Tecnologia, Zona Industrial da Gandra, 4805-017 Barco, Guimarães, Portugal

[2] ICVS/3B's–PT Government Associate Laboratory, Braga/Guimarães, Portugal

* Correspondence: nuno@i3bs.uminho.pt; Tel.: +351-253-510905; Fax: +351-253-510909

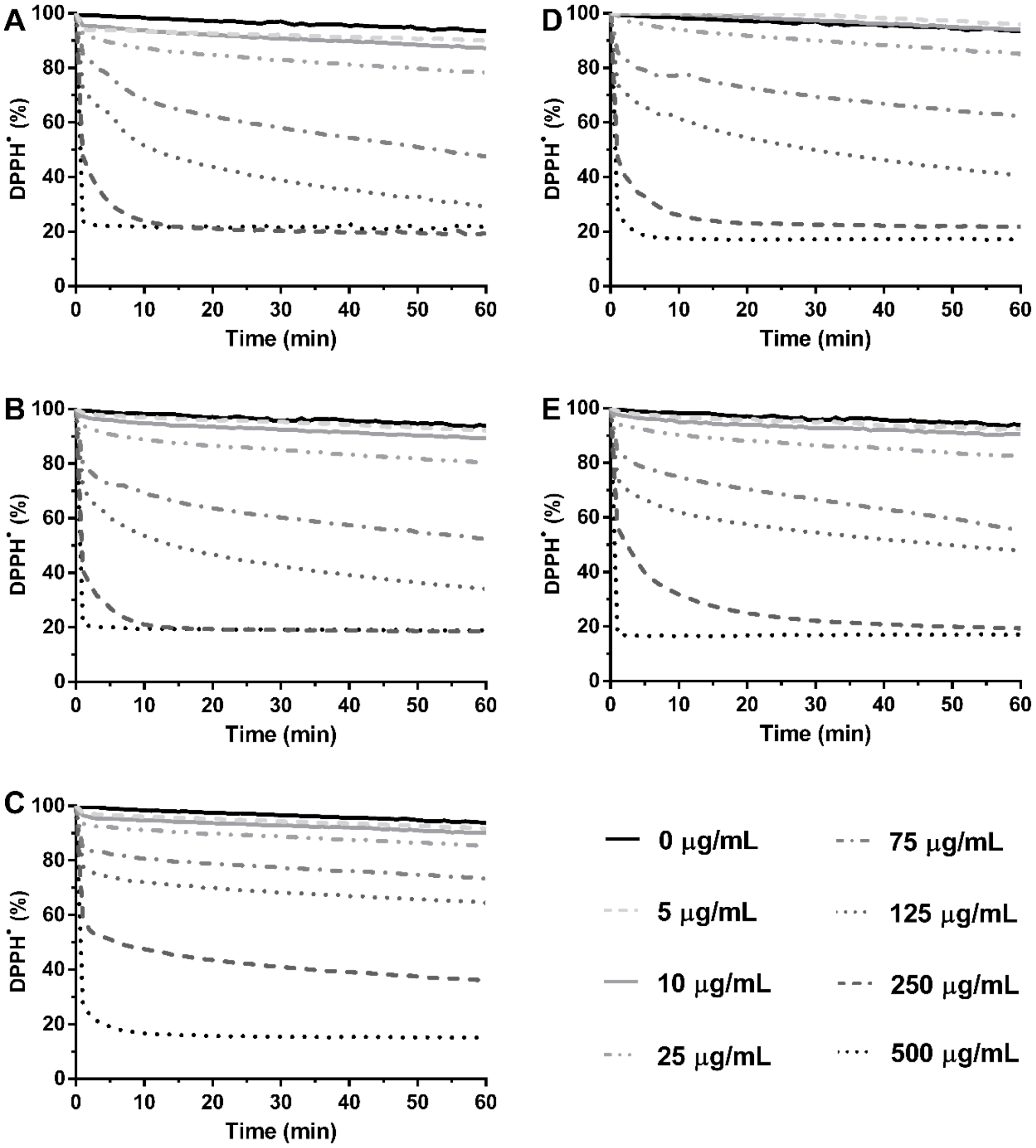

Figure S1 – Representative curves of the antiradical activity of AE-S (**A**), HE-S (**B**), EE-S (**C**), AE-T (**D**), and HE-T (**E**) obtained from *Salvia officinalis* leaves against DPPH•. AE: aqueous extracts; HE: hydroethanolic extracts; EE: ethanolic extracts; S: soxhlet extraction; T: traditional extraction.

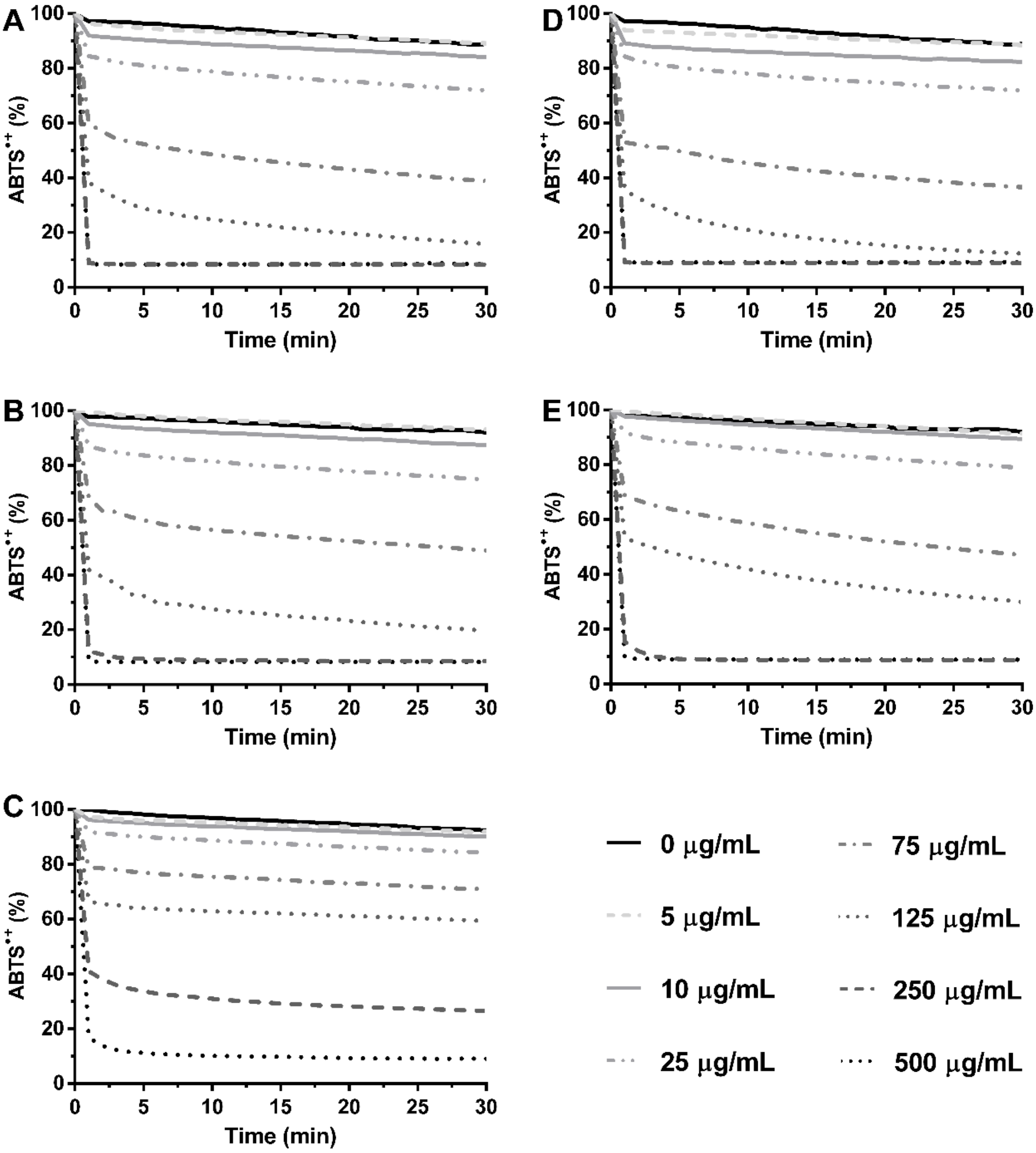

Figure S2 – Representative curves of the antiradical activity of AE-S (**A**), HE-S (**B**), EE-S (**C**), AE-T (**D**), and HE-T (**E**) obtained from *Salvia officinalis* leaves against ABTS$^{\bullet+}$. AE: aqueous extracts; HE: hydroethanolic extracts; EE: ethanolic extracts; S: soxhlet extraction; T: traditional extraction.

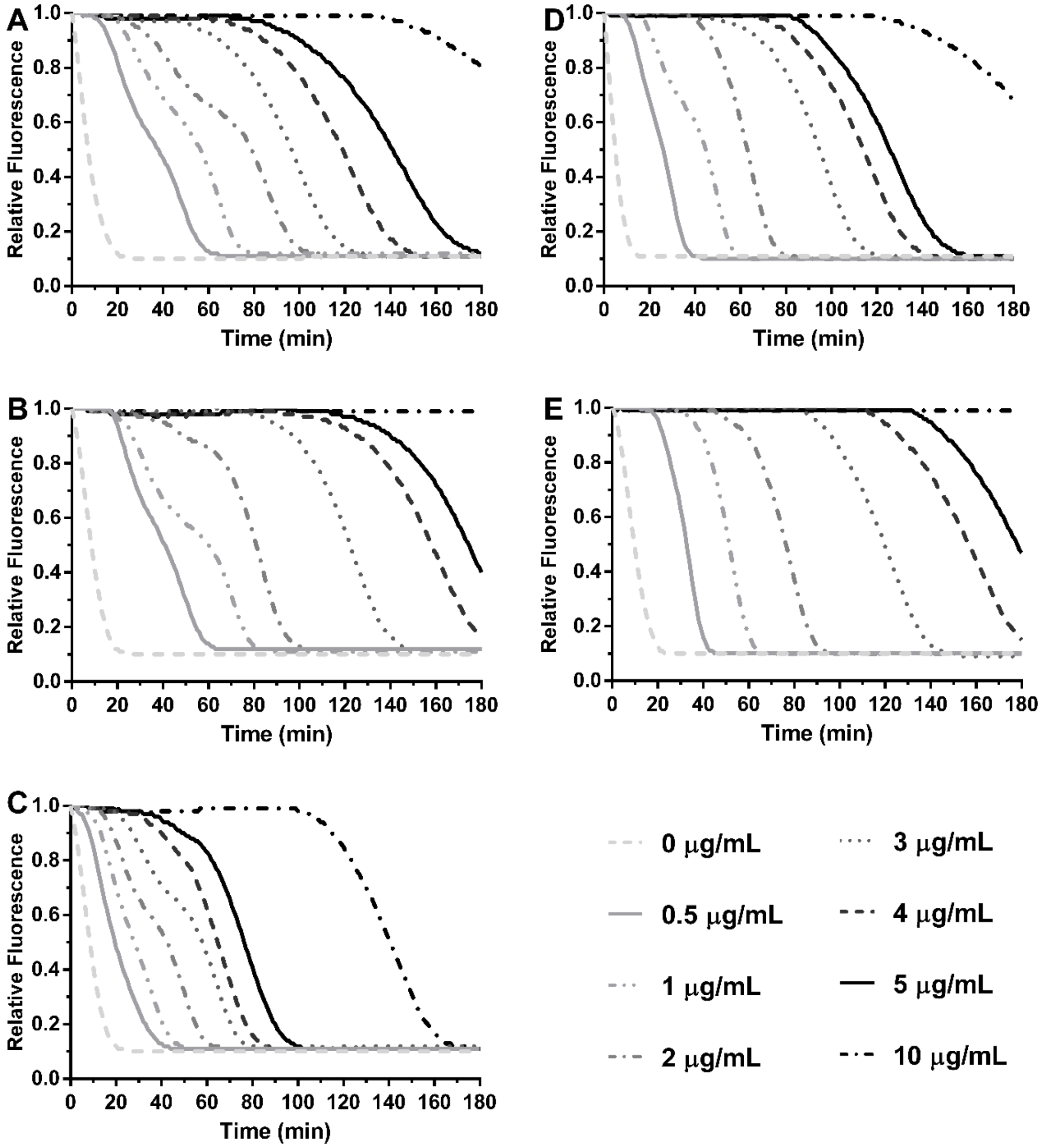

Figure S3 – Representative curves of antioxidant activity of AE-S (**A**), HE-S (**B**), EE-S (**C**), AE-T (**D**), and HE-T (**E**) obtained from *Salvia officinalis* leaves against ROO•. AE: aqueous extracts; HE: hydroethanolic extracts; EE: ethanolic extracts; S: soxhlet extraction; T: traditional extraction.

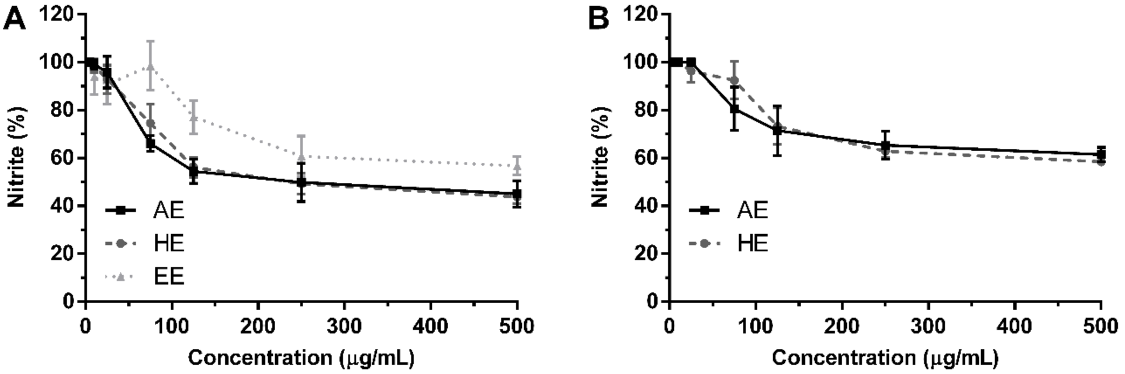

Figure S4 – Antioxidant activity of AE-S, HE-S, EE-S (**A**), AE-T and HE-T (**B**) obtained from *Salvia officinalis* leaves against NO•. AE: aqueous extracts; HE: hydroethanolic extracts; EE: ethanolic extracts; S: soxhlet extraction; T: traditional extraction.

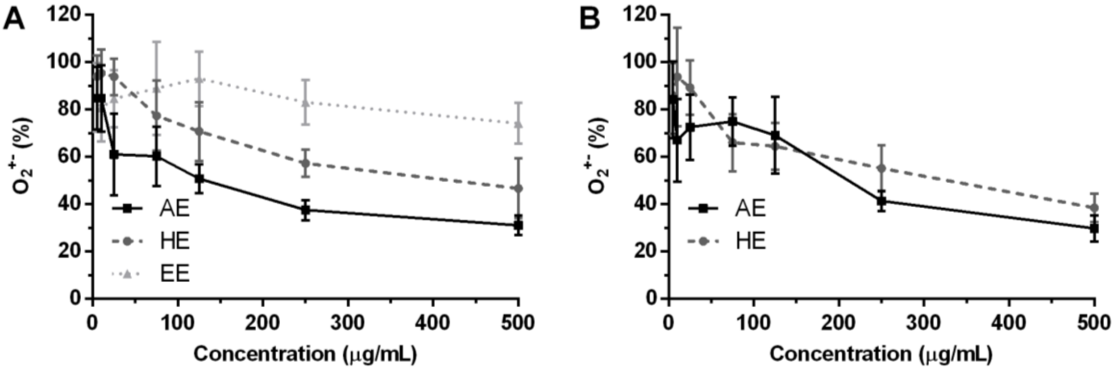

Figure S5 – Antioxidant activity of AE-S, HE-S, EE-S (**A**), AE-T and HE-T (**B**) obtained from *Salvia officinalis* leaves against $O_2^{\bullet -}$. AE: aqueous extracts; HE: hydroethanolic extracts; EE: ethanolic extracts; S: soxhlet extraction; T: traditional extraction.

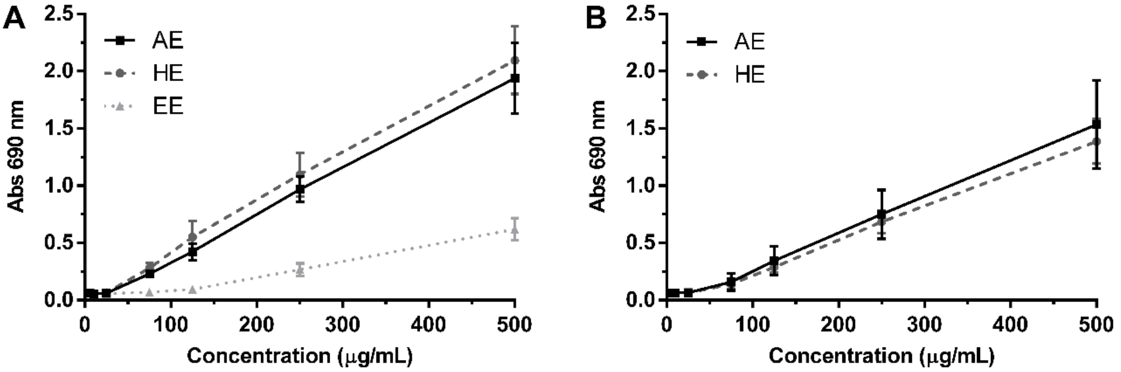

Figure S6 – Reducing power of AE-S, HE-S, EE-S (**A**), AE-T and HE-T (**B**) obtained from *Salvia officinalis* leaves. AE: aqueous extracts; HE: hydroethanolic extracts; EE: ethanolic extracts; S: soxhlet extraction; T: traditional extraction.

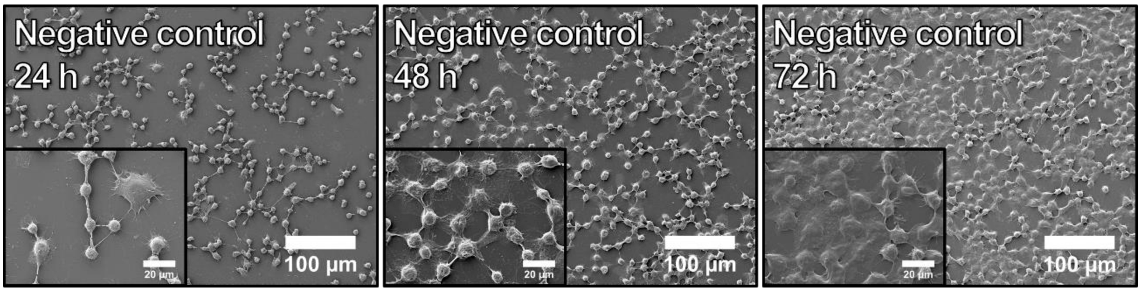

Figure S7 - HR-SEM micrographs of fibroblasts incubated only with medium (negative control) for 24, 48 and 72 h of culture.

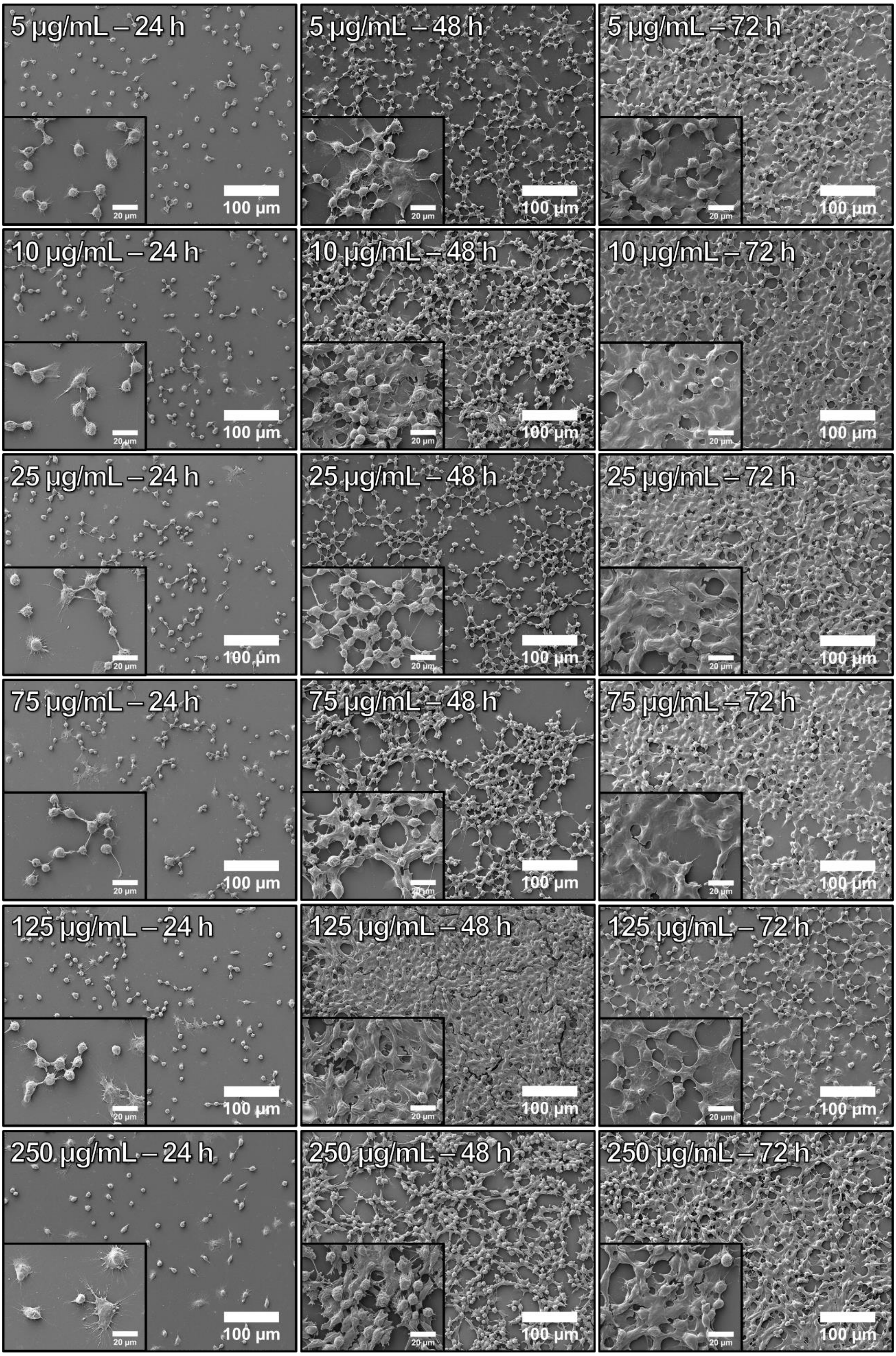

Figure S8 – HR-SEM micrographs of fibroblasts incubated in the presence of *Salvia officinalis* aqueous extracts obtained from soxhlet extraction (AE-S) at different concentrations and culture time. AE: aqueous extracts; S: soxhlet extraction.

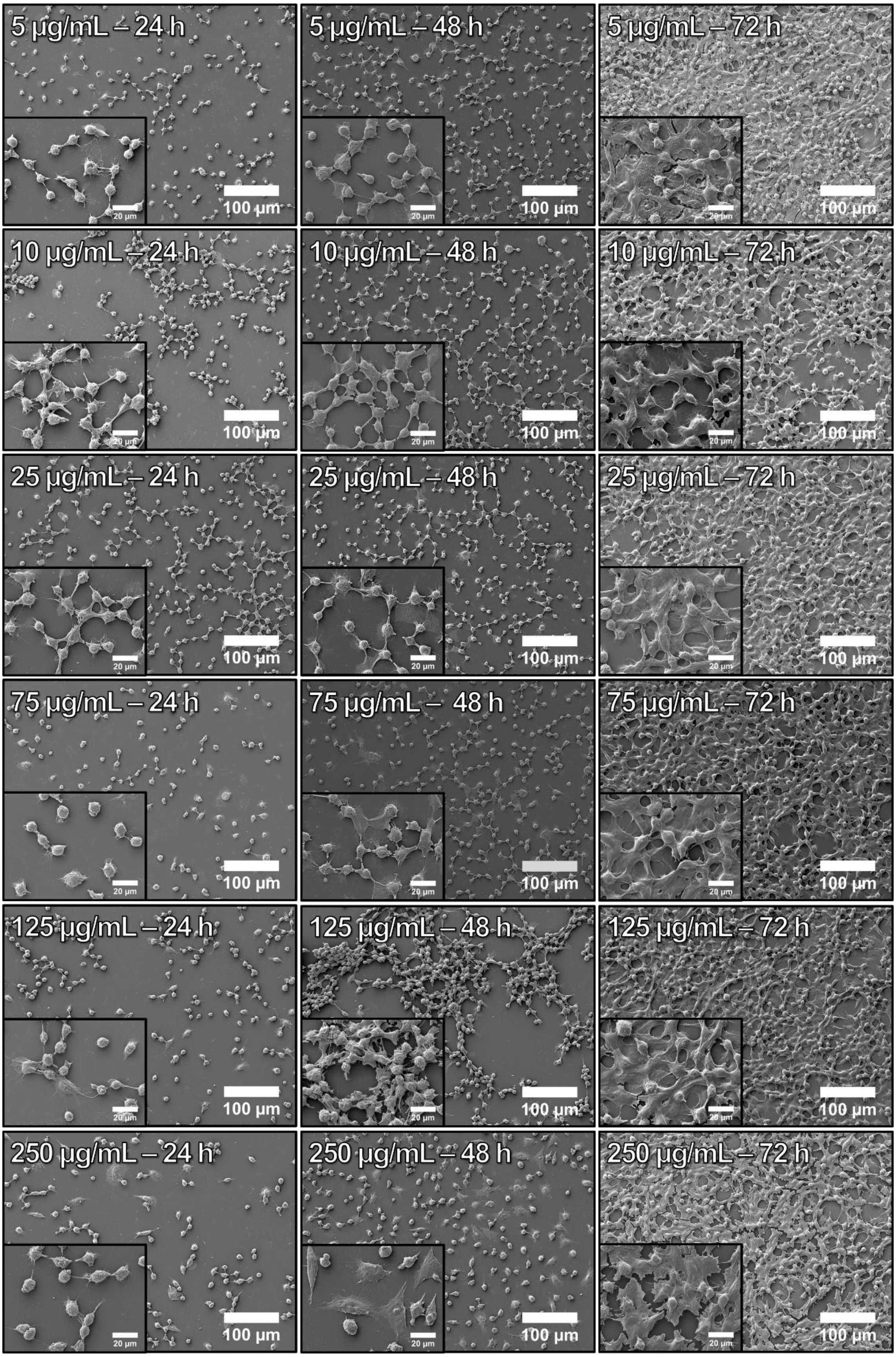

Figure S9 – HR-SEM micrographs of fibroblasts incubated in the presence of *Salvia officinalis* hydroethanolic extracts obtained from soxhlet extraction (HE-S) at different concentrations and culture time. HE: hydroethanolic extracts; S: soxhlet extraction.

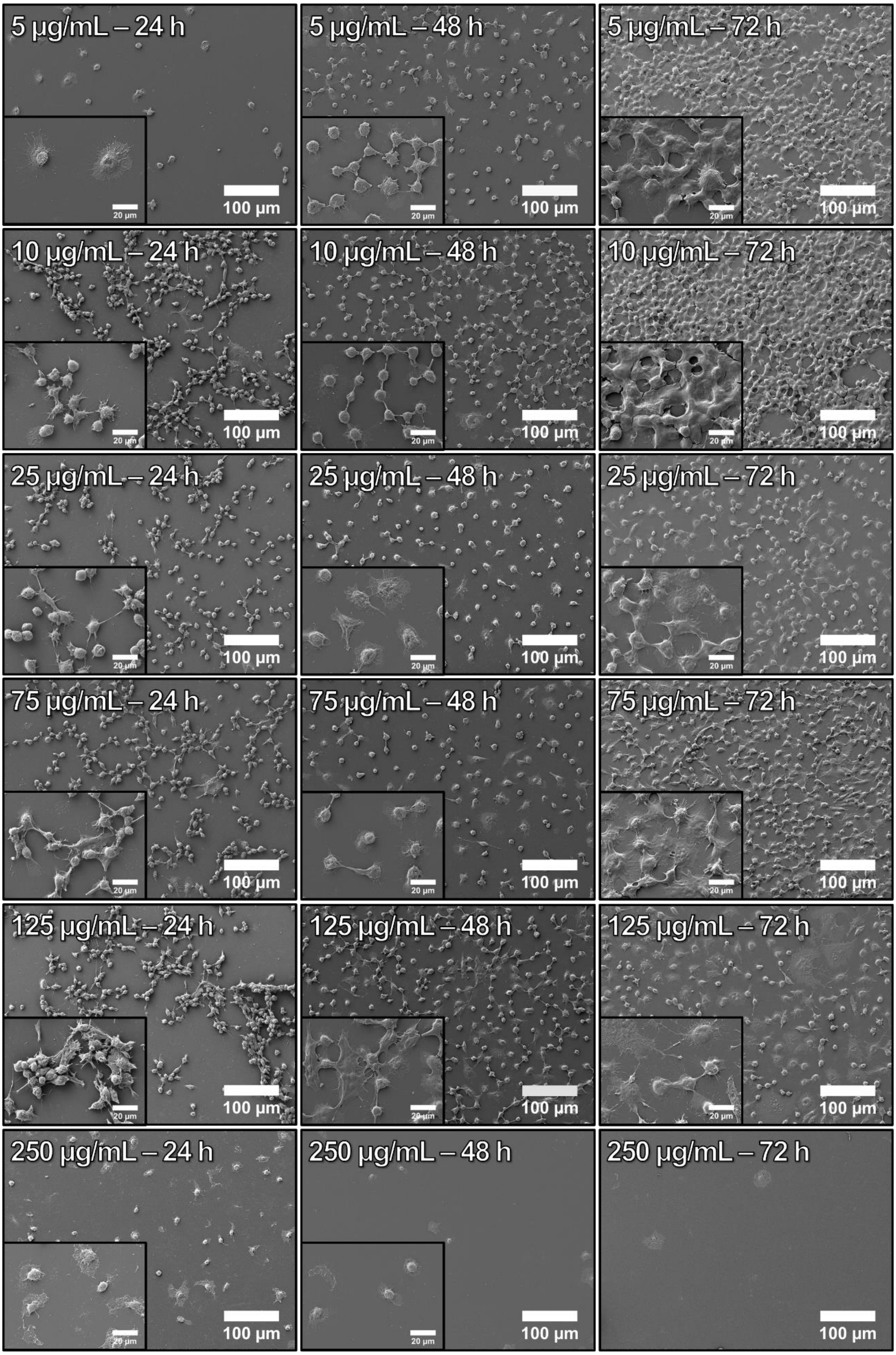

Figure S10 – HR-SEM micrographs of fibroblasts incubated in the presence of *Salvia officinalis* ethanolic extracts obtained from soxhlet extraction (EE-S) at different concentrations and culture time. EE: ethanolic extracts; S: soxhlet extraction.

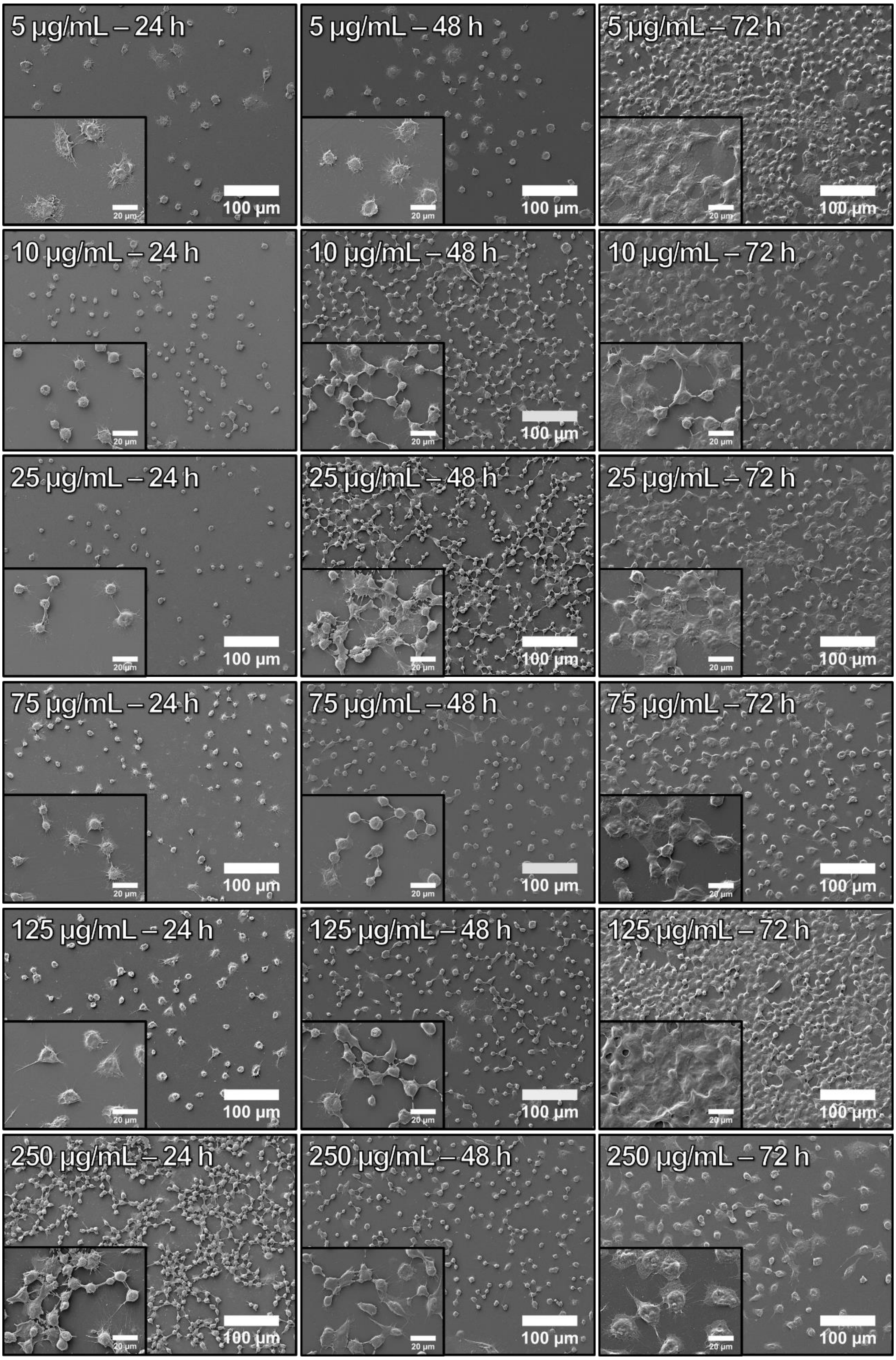

Figure S11 – HR-SEM micrographs of fibroblasts incubated in the presence of *Salvia officinalis* aqueous extracts obtained from traditional extraction (AE-T) at different concentrations and culture time. AE: aqueous extracts; T: traditional extraction.

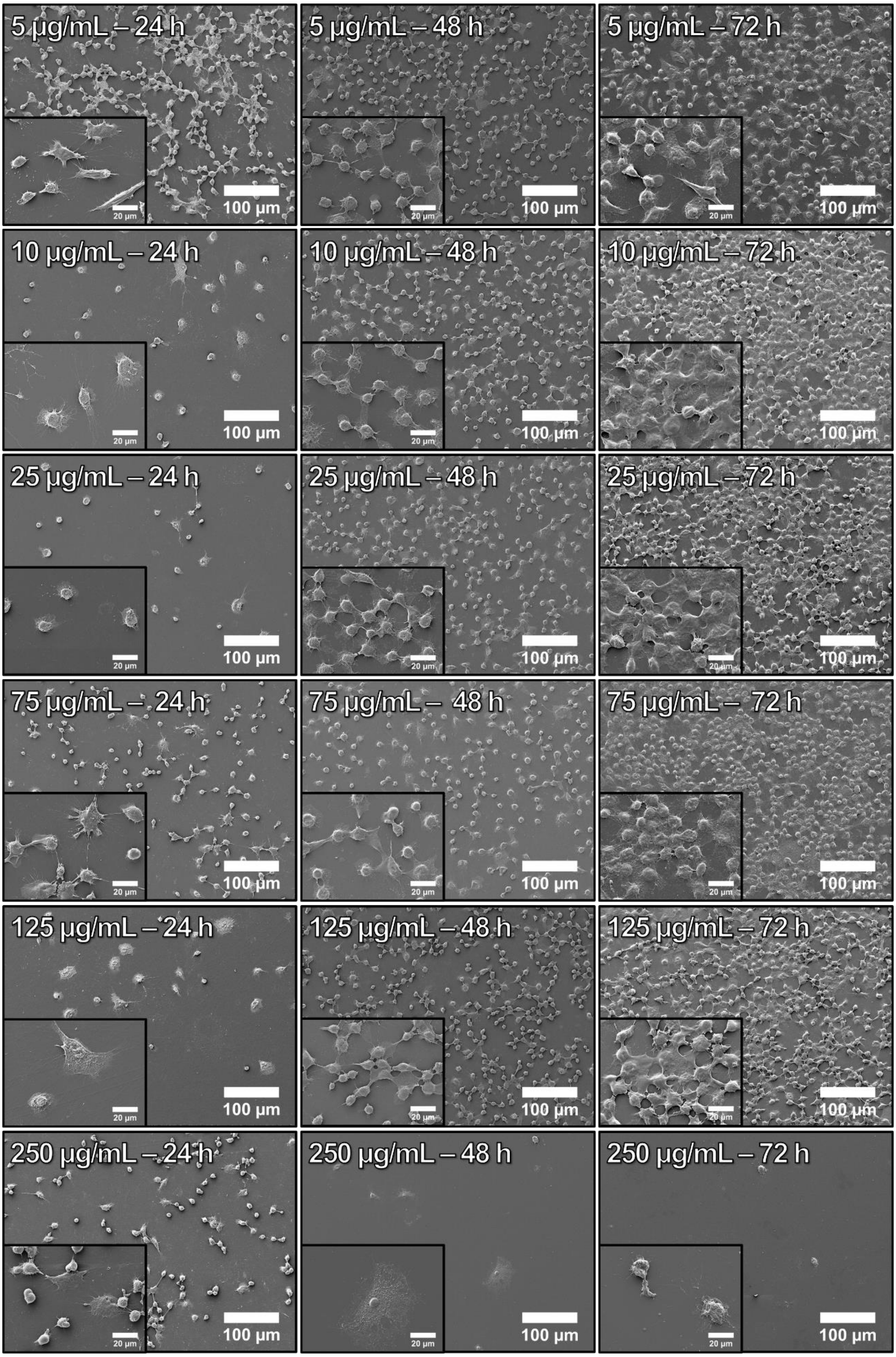

Figure S12 – HR-SEM micrographs of fibroblasts incubated in the presence of *Salvia officinalis* hydroethanolic extracts obtained from traditional extraction (HE-T) at different concentrations and culture time. HE: hydroethanolic extracts; T: traditional extraction.

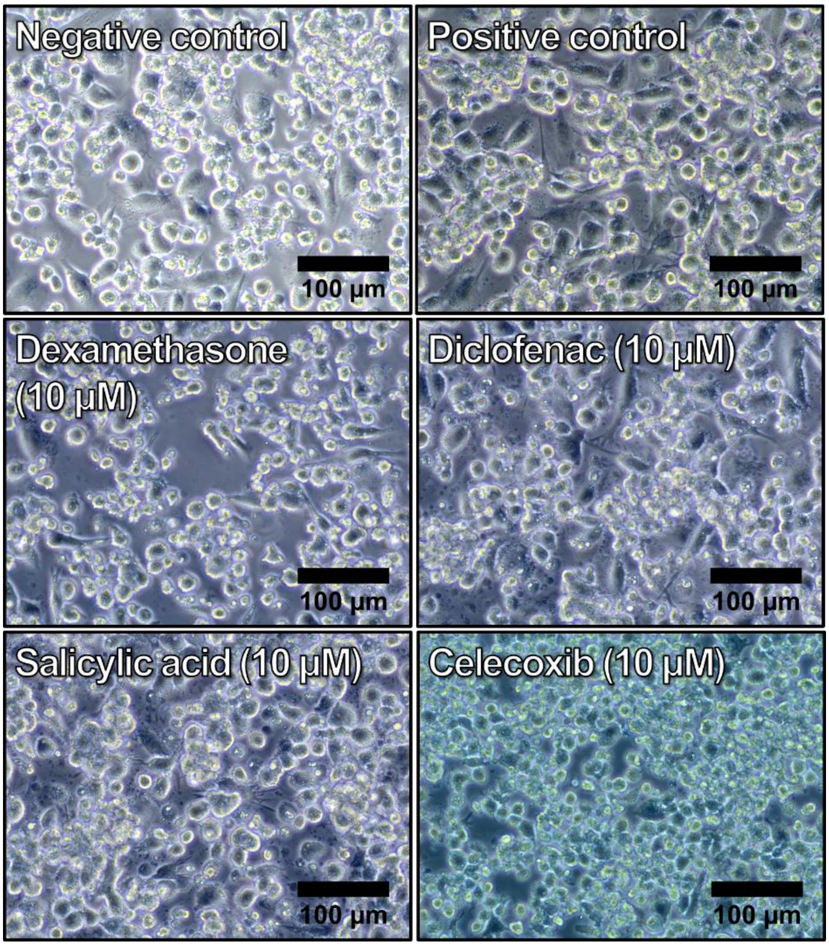

Figure S13 – Optical microphages of non-stimulated macrophages (negative control), LPS-stimulated macrophages (positive control) and LPS-stimulated macrophages cultured in the presence of clinically used anti-inflammatory drugs (dexamethasone, diclofenac, salicylic acid and celecoxib, 10 µM) after 24 h of culture at 37 °C.

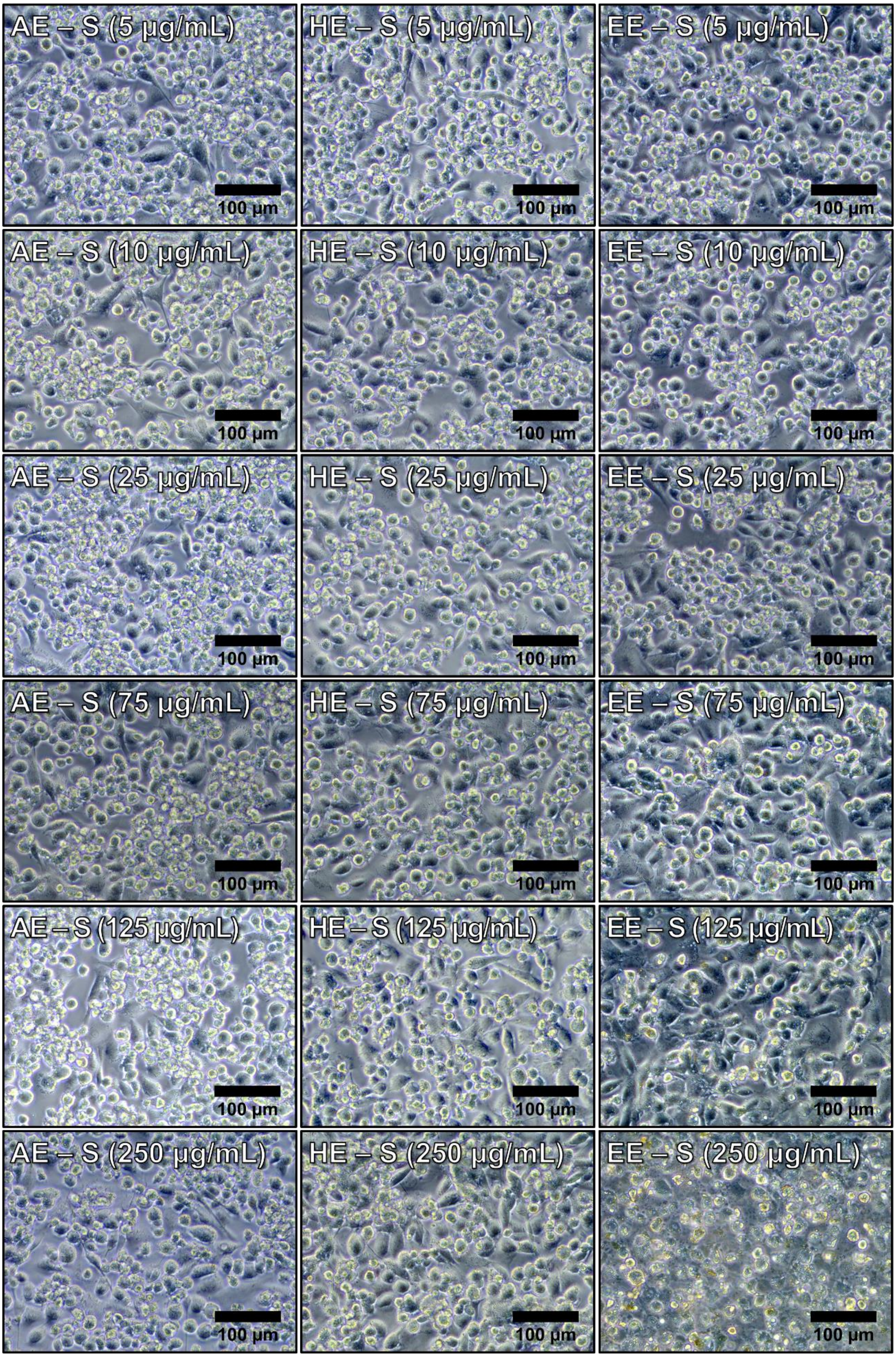

Figure S14 – Optical microphages of non-stimulated macrophages cultured in the presence of AE-S, HE-S, and EE-S obtained from *Salvia officinalis* leaves at different concentrations and culture time. AE: aqueous extracts; HE: hydroethanolic extracts; EE: ethanolic extracts; S: soxhlet extraction.

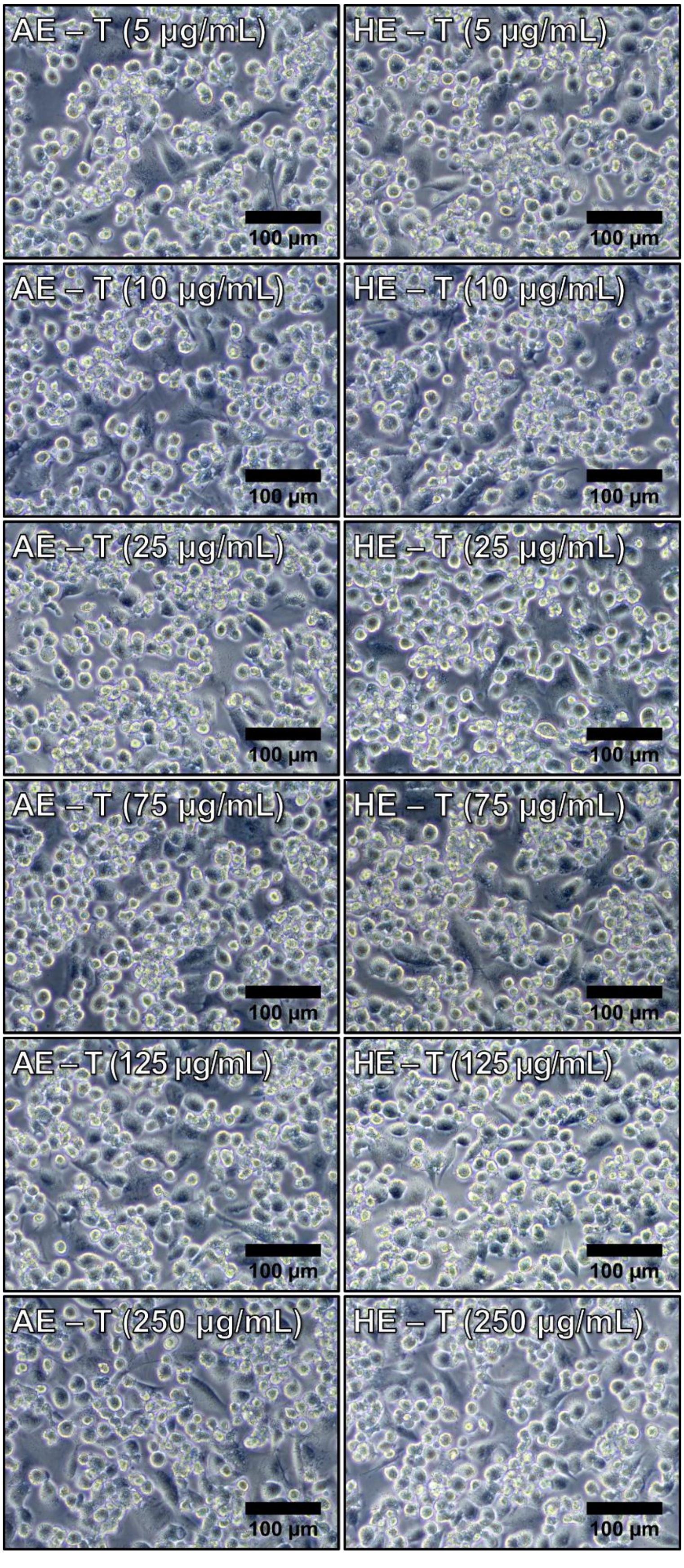

Figure S15 - Optical microphages of non-stimulated macrophages cultured in the presence of AE-T and HE-T obtained from *Salvia officinalis* leaves at different concentrations and culture time. AE: aqueous extracts; HE: hydroethanolic extracts; T: traditional extraction.

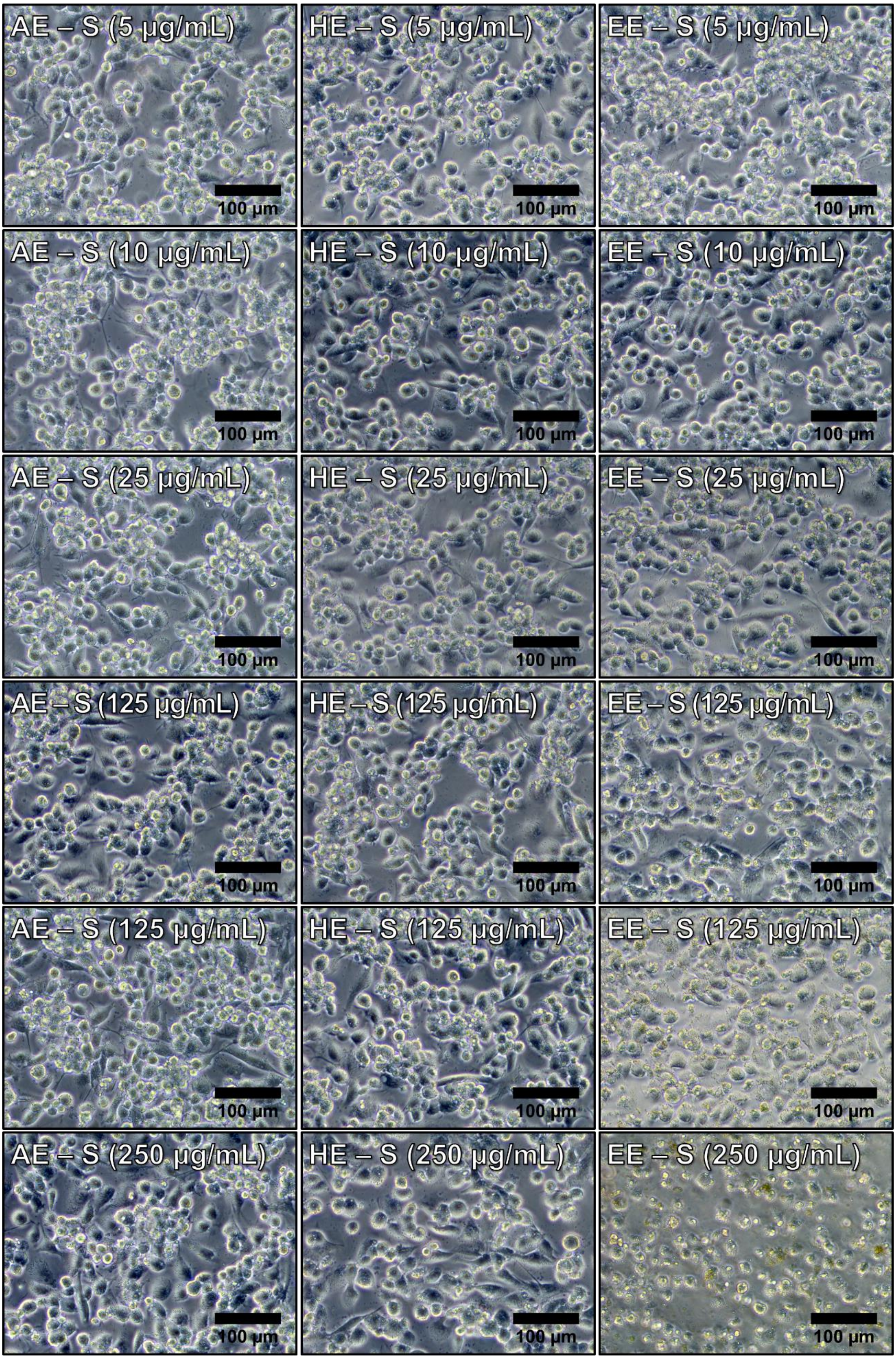

Figure S161 – Optical microphages of LPS-stimulated macrophages cultured in the presence of AE-S, HE-S, and EE-S obtained from *Salvia officinalis* leaves at different concentrations and culture time. AE: aqueous extracts; HE: hydroethanolic extracts; EE: ethanolic extracts; S: soxhlet extraction.

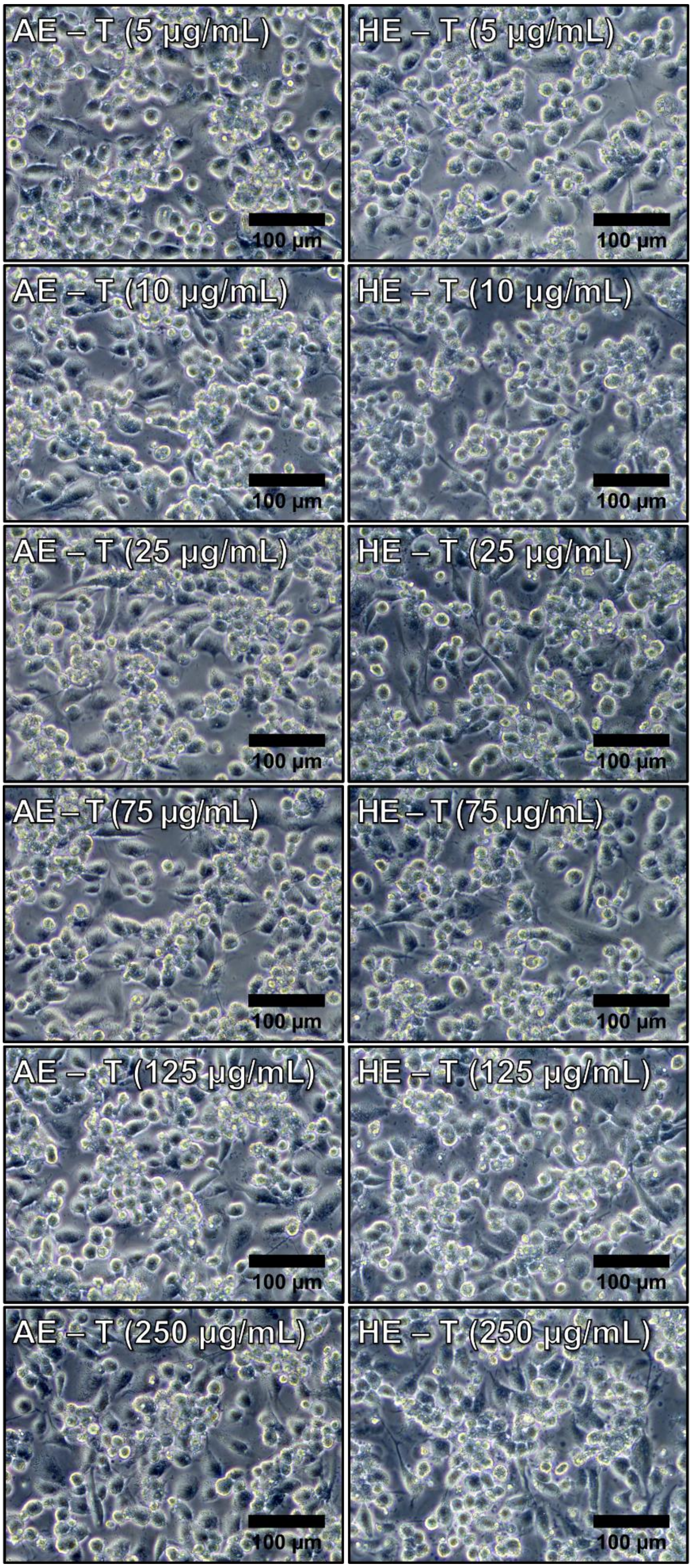

Figure S172 – Optical microphages of non-stimulated macrophages cultured in the presence of AE-T and HE-T obtained from *Salvia officinalis* leaves at different concentrations and culture time. AE: aqueous extracts; HE: hydroethanolic extracts; T: traditional extraction.
